# Excess diacylglycerol at the endoplasmic reticulum disrupts endomembrane homeostasis and autophagy

**DOI:** 10.1186/s12915-020-00837-w

**Published:** 2020-08-28

**Authors:** Dan Li, Shu-Gao Yang, Cheng-Wen He, Zheng-Tan Zhang, Yongheng Liang, Hui Li, Jing Zhu, Xiong Su, Qingqiu Gong, Zhiping Xie

**Affiliations:** 1 State Key Laboratory of Microbial Metabolism & Joint International Research Laboratory of Metabolic and Developmental Sciences, School of Life Sciences and Biotechnology, Shanghai Jiao Tong University, #800 Dong-Chuan Road, Shanghai, 200240 People’s Republic of China; 2 School of Biology and Basic Medical Sciences, Soochow University, Suzhou, Jiangsu People’s Republic of China; 3 College of Life Sciences, Nanjing Agricultural University, Nanjing, Jiangsu People’s Republic of China

**Keywords:** Phospholipid, Glycerolipid, Intracellular trafficking, Organelle, Autophagy

## Abstract

**Background:**

When stressed, eukaryotic cells produce triacylglycerol (TAG) to store nutrients and mobilize autophagy to combat internal damage. We and others previously reported that in yeast, elimination of TAG synthesizing enzymes inhibits autophagy under nitrogen starvation, yet the underlying mechanism has remained elusive.

**Results:**

Here, we show that disruption of TAG synthesis led to diacylglycerol (DAG) accumulation and its relocation from the vacuolar membrane to the endoplasmic reticulum (ER). We further show that, beyond autophagy, ER-accumulated DAG caused severe defects in the endomembrane system, including disturbing the balance of ER-Golgi protein trafficking, manifesting in bulging of ER and loss of the Golgi apparatus. Genetic or chemical manipulations that increase consumption or decrease supply of DAG reversed these defects. In contrast, increased amounts of precursors of glycerolipid synthesis, including phosphatidic acid and free fatty acids, did not replicate the effects of excess DAG. We also provide evidence that the observed endomembrane defects do not rely on Golgi-produced DAG, Pkc1 signaling, or the unfolded protein response.

**Conclusions:**

This work identifies DAG as the critical lipid molecule responsible for autophagy inhibition under condition of defective TAG synthesis and demonstrates the disruption of ER and Golgi function by excess DAG as the potential cause of the autophagy defect.

## Background

Lipids are essential building blocks of life. Polar lipids, including phospholipids, sphingolipids, and sterols, are major constituents of cellular membranes. Neutral lipids, in particular TAG, are employed as a storage medium of carbon nutrients [1, 2]. Storage lipid synthesis is induced not only in the presence of nutrient surplus, but also in response to stress [3–6]. However, the amount of lipids that a cell or an organism can utilize and tolerate is limited. Too much lipid, from either environmental intake or de novo synthesis, can disrupt cellular homeostasis, leading to ER stress, ROS production, and cell death [7–9].

Recent studies revealed a close association of lipid metabolism with autophagy. Autophagy is a stress response pathway that utilizes double-membrane autophagosomes to clean up obsolete intracellular materials in eukaryotes [10, 11]. Autophagosomes are formed from precursor membrane sacs, known as phagophores or isolation membranes. The phagophore is thought to emerge from a specialized region of ER [12–14]. The close contact between the phagophore and the ER facilitates lipid channeling by lipid transfer proteins [15, 16]. Furthermore, anterograde trafficking out of the ER via COPII-coated vesicles is essential for autophagy [17–19]. This and many other vesicular trafficking pathways are reprogramed under stress conditions to connect the autophagy machinery with the rest of the endomembrane system, including the ER, Golgi, and endocytic compartments [20, 21]. Finally, the formation of autophagosomes appears to be coordinated with localized lipid biosynthesis, linking membrane dynamics with primary metabolism [22–24].

In yeast *Saccharomyces cerevisiae*, autophagy is strongly induced by nitrogen starvation [25]. Under the same condition, yeast cells continue glucose consumption and upregulate TAG production [26], which is analogous to observations in starved mammalian cells [27, 28]. The last step in TAG synthesis is catalyzed by two acyltransferases, Dga1 and Lro1 (Additional file 1: Fig. S1A) [29–31]. We and others have previously shown that elimination of both enzymes led to severe inhibition of autophagy [26, 32–34]. Different models have been proposed to explain this phenomenon, mainly from the perspective of fatty acid (FA) supply. In the present study, we set out to answer two key questions: first, what is the nature of the autophagy defect, and second, whether the disruption can be traced to a single causal factor while being consistent with established data.

## Results

### Block of TAG synthesis disrupts the endomembrane system

**Fig. 1:**
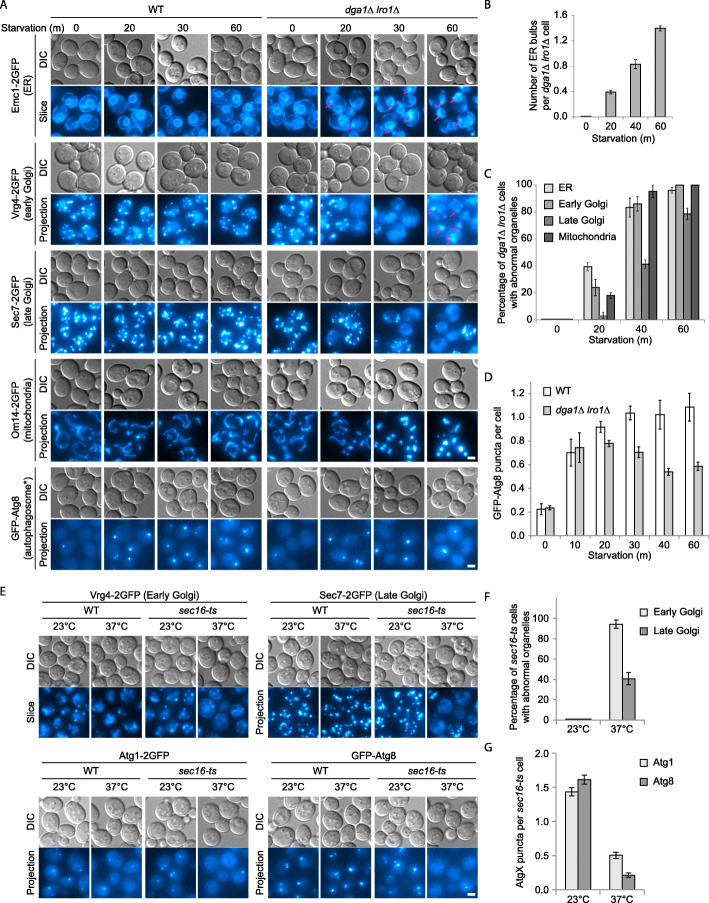
Block in TAG synthesis disrupts the endomembrane system. **a** Starvation triggers alterations in the ER, Golgi, and mitochondrial morphology in cells defective in TAG synthesis (*dga1Δ lro1Δ*). Cells expressing indicated organelle markers were transferred from rich medium to nitrogen starvation medium. Organelle morphology was observed by fluorescent microscopy at the indicated time points. Representative images from three independent repeats are shown. DIC, differential interference contrast; Slice, a single slice in the fluorescence z-stack; Projection, max intensity projection of the fluorescence z-stack. Autophagosome*, complete or incomplete autophagosomal structure. Arrows, bulbous structures on the ER. Scale bar, 2 μm. **b–d** Quantification of organelle defects in **a**. **b** Number of ER bulbs per cell. **c** Percentage of cells displaying abnormal organelle morphology (ER bulb formation, Golgi disappearance, mitochondrial fragmentation). **d** Progression of autophagy defect as indicated by the decline in the number of GFP-Atg8 dots. Error bar, standard deviation, *n* = 3. **e** Inhibition of ER exit leads to disappearance of Golgi and impairment of Atg protein recruitment. *sec16-ts* cells expressing indicated organelle markers were first grew to mid-log phase under permissive temperature, then transferred to nitrogen starvation medium and incubated under either permissive temperature or non-permissive temperature for 1 h. Images presented as in **a**. **f** Quantification of Golgi defects in **e**. Error bar, standard deviation, *n* = 3. **g** Quantification of Atg1 and Atg8 recruitment defects in **e**. Error bar, standard deviation, *n* = 3

**Fig. 2:**
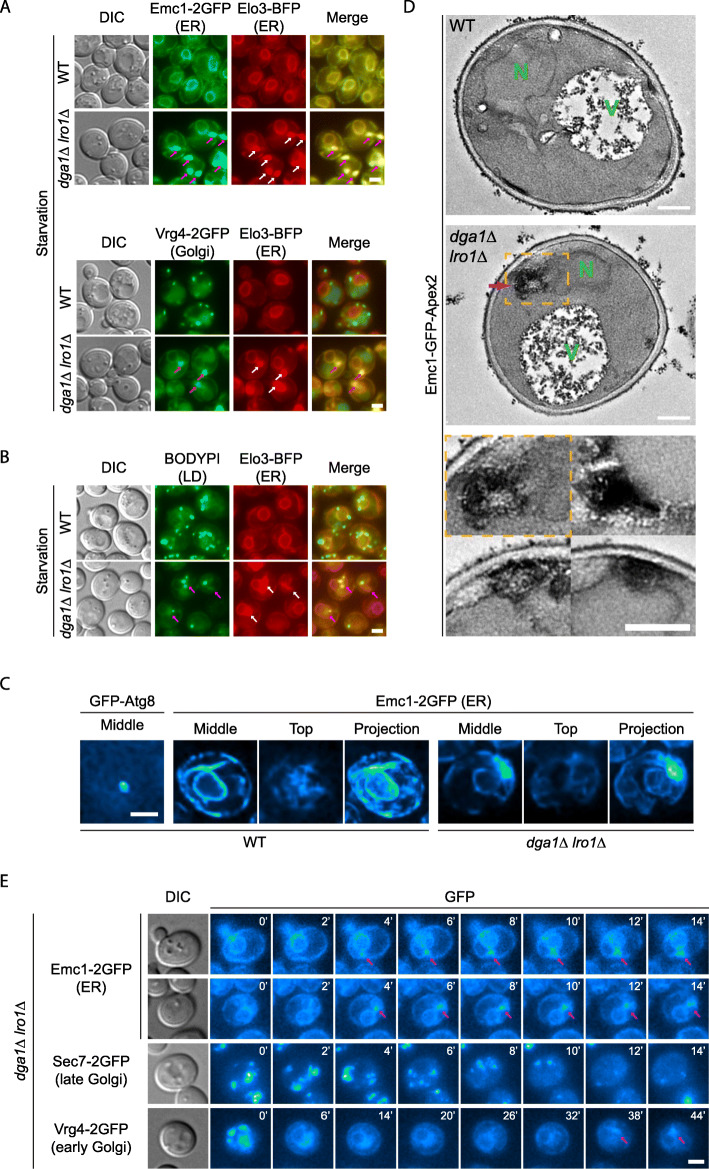
Characterization of endomembrane system defects in TAG production defective cells. **a** The bulbous ER structure contains multiple ER and Golgi proteins. Cells co-expressing two protein chimeras as indicated were starved for 1 h. Representative image slices from individual channels and merged channels are shown. Arrows, bulbous structures on the ER. Scale bar, 2 μm. **b** The bulbous ER structures are not lipid droplets. Cells expressing Elo3-BFP were starved for 1 h and stained with BODIPY. Images presented as in **a**. **c** The ER bulbs are not hollow vesicles. Cells were starved for 1 h. Autophagosomes and ER bulbs were imaged using the Super Resolution via Optical Re-assignment (SORA) technique. Scale bar, 2 μm. **d** Electron micrograph of yeast cells carrying the indicated genotypes. Cells expressing Emc1-GFP-Apex2 were starved for 1 h and stained with DAB. Representative transmission electron micrographs from two independent repeats are shown. N, nucleus. V, vacuoles. Purple arrows, electron-dense structures connected to the ER. Inserts below, magnified view of demarcated area above and three additional areas from *dga1Δ lro1Δ* samples, showing the electron-dense structures. Scale bar, 0.5 μm. **e** Time-lapse imaging of the endomembrane defects. *dga1Δ lro1Δ* cells were transferred from rich medium to nitrogen starvation medium. Representative image slices (Emc1) or projections (Sec7, Vrg4) at the indicated time point are shown. Time 0 corresponds to 20 min after the medium transfer. Scale bar, 2 μm

As the process of autophagy relies on membrane and protein input from the rest of the endomembrane system through direct contacts and vesicular trafficking pathways [20], we first analyzed the morphology of major membrane-based organelles in order to understand the nature of autophagy defect in *dga1Δ lro1Δ* cells (Fig. 1a–c, Additional file 1: Fig. S2). Markers of peroxisomes, late endosomes, and vacuoles displayed distribution patterns comparable to those in wild-type cells (Additional file 1: Fig. S2). In contrast, we noticed substantial alterations in the ER, Golgi, and mitochondria (Fig. 1a–c, Additional file 1: Fig. S2). As *dga1Δ lro1Δ* cells entered nitrogen starvation, bulbous structures sprouted from the ER, the multi-puncta pattern of Golgi marker proteins vanished, and mitochondria became fragmented. The same changes were observed using multiple marker proteins of the same organelles (Fig. 1a, Additional file 1: Fig. S2), which suggest that an overall alteration in the conditions of the labeled organelles, rather than that of individual proteins, occurred in *dga1Δ lro1Δ* cells. The changes in organelle morphology happened progressively. The pace of these changes coincided with the occurrence of autophagy inhibition, as indicated by the reduction in the number of GFP-Atg8 positive autophagic structures (Fig. 1a, d). Colocalization analysis revealed that the bulbs contained both ER and Golgi proteins (Fig. 2a). They were not strongly stained by BODIPY (Fig. 2b), indicating that the bulbs are separate from lipid droplets, which also frequently associate with the ER. The size of most bulbs was visibly larger than that of regular autophagosomes. In live cell super resolution microscopy, we could easily see ring-like structure in wild-type cells expressing GFP-Atg8. Under the same condition, few ER bulbs in *dga1Δ lro1Δ* cells displayed hollow cavities (Fig. 2c), implying that the structures contain membrane proteins and potentially membrane structures inside. We further characterized the ER bulbs by transmission electron microscopy (TEM). In those samples, ER was labeled by an Apex2 chimera and stained with diaminobenzidine (DAB) [35]. In *dga1Δ lro1Δ* cells, we observed membrane-like electron-dense structures on or connected to the ER (Fig. 2d). The electron-dense structures were absent in wild-type cells, implying that they correspond to the bulbous structures seen under light microscopy. In time-lapse imaging, the bulbous structures appeared to emerge from the ER (Fig. 2e). For Golgi proteins that go back to the ER, their translocation to the regular ER network (both nuclear and peripheral pool) occurred first, in a time period that corresponds to that of ER bulb emergence (Fig. 2e). The appearance of Golgi proteins in the ER bulbs occurred much later, close to 1 h after starvation (Fig. 2e), which implies that the presence of Golgi proteins at the bulbs is secondary to their regression into the ER. These results demonstrate that the impact of blocking TAG synthesis is not limited to the autophagy pathway. It led to significant disturbances in the endomembrane system, disrupting normal ER-Golgi protein trafficking.

We previously reported that the autophagy defect in *dga1Δ lro1Δ* cells was characterized by reduced recruitment of Atg8, Atg1, and Atg5, but not the scaffold proteins, to the phagophore assembly site (PAS) [26]. To examine the relationship between endomembrane alterations and autophagy, we experimented with blocking ER protein export using a temperature-sensitive mutant of *SEC16*. Upon shifting to non-permissive temperature, *sec16-ts* cells gradually lost punctate distribution of Golgi marker proteins (Fig. 1e, f), partially reproducing the endomembrane alteration seen in *dga1Δ lro1Δ* cells. Inactivation of Sec16 also reduced PAS recruitment of Atg8 and Atg1 (Fig. 1e, g). This result is consistent with ER-Golgi trafficking defect being a major contributor to the autophagy defect in *dga1Δ lro1Δ* cells.

### Endomembrane defects in TAG production defective cells are caused by the accumulation of an intermediate metabolite

Regardless of the complexity of potential downstream signaling, the effect of blocking a biochemical reaction often stems from alterations in the levels of its upstream metabolites, downstream metabolites, or a combination thereof. Here, we took a stepwise approach to pinpoint the critical metabolite causing the endomembrane and autophagy disruptions. First, we analyzed mutants lacking enzymes potentially involved in TAG hydrolysis or product utilization [32] and found no substantial alterations in autophagic flux, as measured by the pho8Δ60 assay and GFP-Atg8 processing assay (Additional file 1: Fig. S1B-C). We and others have previously shown that the well-established TAG lipases, Tgl3 and Tgl4, are not essential for autophagy [26, 32]. Concluding that autophagy does not require TAG utilization, we focused our remaining analysis on upstream reactions leading to or diverting from TAG synthesis.

**Fig. 3:**
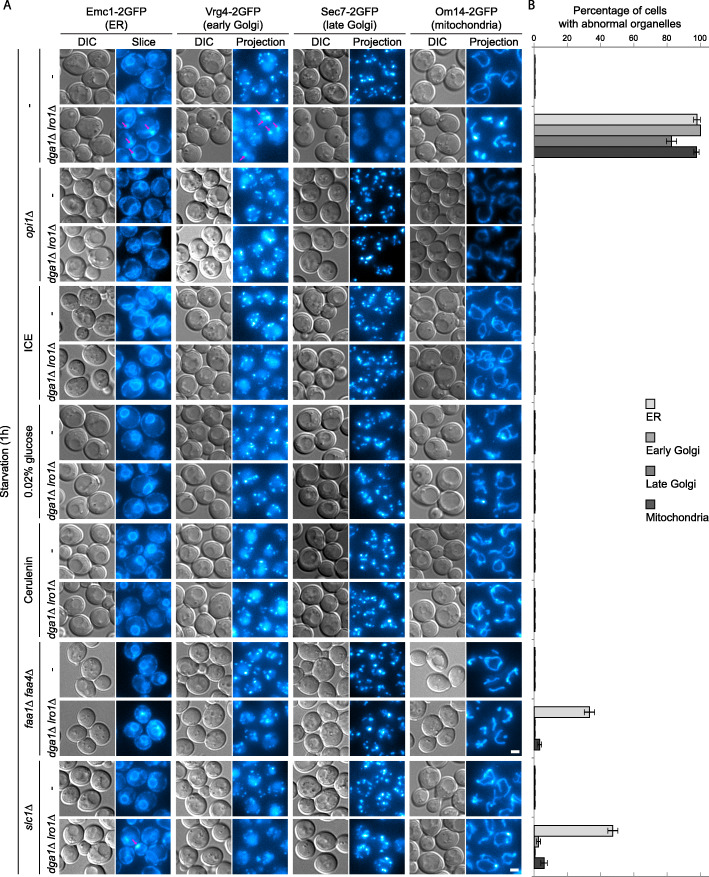
Upregulating phospholipid synthesis or constraining precursor influx rescues endomembrane defects in TAG production defective cells. **a**, **b** Phospholipid production was upregulated by (1) knocking out *OPI1* or (2) supplying key reactants (inositol, choline, and ethanolamine/ICE). Precursor influx was constrained by (1) 100-fold reduction of glucose supply (0.02% glucose), (2) chemical inhibition of fatty acid synthase (cerulenin), (3) elimination of major fatty acyl-CoA synthetases (*faa1Δ faa4Δ*), or (4) elimination of a key lysoPA acyltransferase (*slc1Δ*). **a** Representative images presented as in Fig. 1a. Scale bar, 2 μm. **b** Quantification of cells displaying organelle defects. Error bar, standard deviation, *n* = 3

**Fig. 4:**
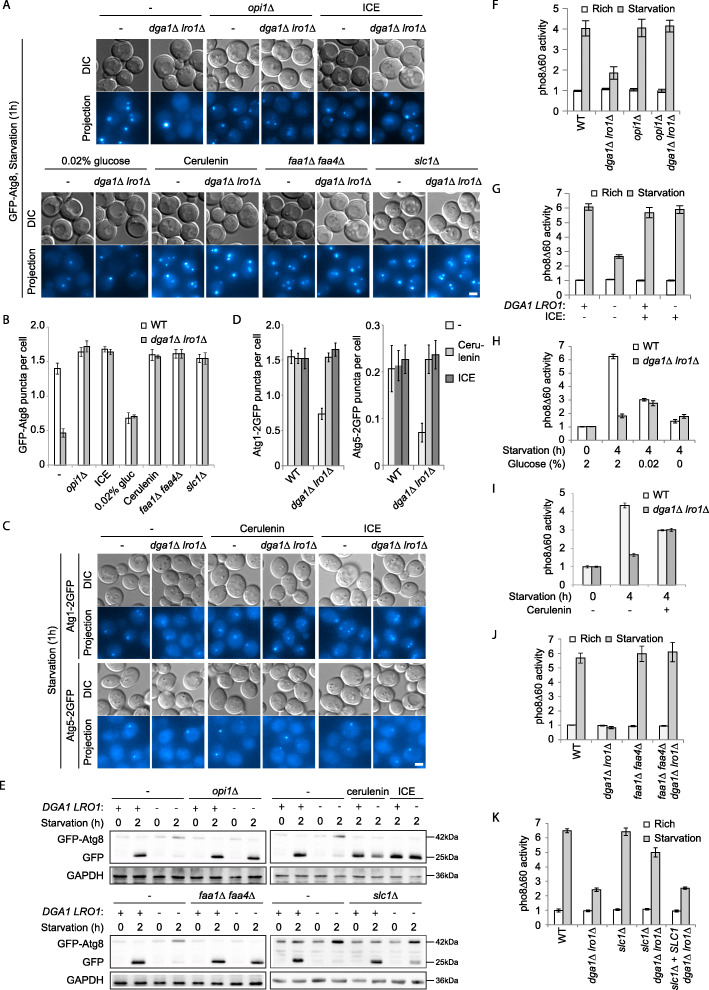
Upregulating phospholipid synthesis or constraining precursor influx restores autophagy in TAG production defective cells. Autophagy was assessed by formation of GFP-Atg8 puncta (**a**, **b**), formation of Atg1 and Atg5 puncta (**c**, **d**), proteolytic processing of GFP-Atg8 (**e**), and pho8Δ60 assay (**f–k**). Induction of phospholipid production and reduction of precursor influx were achieved as in Fig. 3. **a**, **c** Representative microscopy images presented as in Fig. 1a. Cells starved for 1 h. **b**, **d** Quantification of Atg8, Atg1, and Atg5 puncta per cell in **a**, **c**. Error bar, standard deviation, *n* = 3. **e** Representative immunoblots from three independent repeats. Cells starved for 2 h. **f–k** pho8Δ60 enzymatic assay. Cells starved for 4 h. Error bar, standard deviation, *n* = 3

TAG and phospholipids are both glycerolipids and share early stages of their biosynthesis up to phosphatidic acid (PA) and diacylglycerol (DAG) (Additional file 1: Fig. S1A). We found that upregulation of phospholipid synthesis, either by relieving transcriptional repression of the enzymes (*opi1Δ*) or by addition of reactants (inositol, choline, and ethanolamine/ICE) [31], led to restoration of Golgi and mitochondria morphology (Fig. 3a, b). The ER morphology was largely restored with only sporadic bulbous structures, but with a visible expansion of non-nuclear ER membranes (Fig. 3a, b). Such expansion has been associated with upregulation of phospholipid biosynthesis previously [34, 36]. Among the reactants tested, choline alone was able to ameliorate the defects significantly (data not shown); however, the addition of all three (ICE) led to maximal recovery. Autophagy in *dga1Δ lro1Δ* was also recovered by *opi1Δ* and ICE supplementation, as indicated by the formation of GFP-Atg8 puncta, GFP-Atg8 processing, and pho8Δ60 enzymatic activation (Fig. 4a, b, e–g). Furthermore, the characteristic Atg1 and Atg5 recruitment defect was reverted by the addition of ICE (Fig. 4c, d).

Next, we took several approaches to decrease carbon influx towards glycerolipid synthesis, including (1) 100-fold reduction of glucose supply (0.02% glucose), (2) chemical inhibition of fatty acid synthase (cerulenin), (3) elimination of major fatty acyl-CoA synthetases (*faa1Δ faa4Δ*), and (4) elimination of one of the two key lysoPA acyltransferases (*slc1Δ*) [31, 37]. With the first two approaches, the morphologies of ER, Golgi, and mitochondria in *dga1Δ lro1Δ* cells remained normal upon starvation (Fig. 3a, b). For *faa1Δ faa4Δ*, the recovery of ER morphology was partial, possibly because the recycling of free fatty acid by acyl-CoA synthetases only accounts for part of the lipid precursor influx. The partial effect caused by *slc1Δ* is also consistent with the presence of remaining lysoPA acyltransferases. The absolute levels of autophagy became lower with less glucose (Fig. 4a, b, h), possibly reflecting the reliance of autophagy on energy production. Cerulenin addition also reduced total autophagic flux (Fig. 4e, i). Nevertheless, both low glucose and cerulenin caused the levels of autophagy to become comparable between *dga1Δ lro1Δ* and wild-type controls. The PAS recruitment of Atg1 and Atg5 was difficult to assess in low glucose condition due to dim signal (data not shown). The recruitment of these two Atg proteins became normal with cerulenin treatment (Fig. 4c, d). For *faa1Δ faa4Δ*, autophagy was fully restored, as indicated by all three assays (Fig. 4a, b, e, j). For *slc1Δ*, although the number of GFP-Atg8 was recovered (Fig. 4a, b), final autophagic flux as indicated by GFP-Atg8 processing and pho8Δ60 was still partially compromised (Fig. 4e, j).

The approaches we took to reduce glycerolipid synthesis interfered at the steps of glucose consumption, fatty acid synthesis, free fatty acid utilization, and lyso-PA to PA conversion, respectively. With all four approaches being effective at rescuing the phenotypes, these data collectively point to metabolites downstream of PA synthesis as the endomembrane disrupter. Combined with the fact that diverting PA and DAG towards phospholipid synthesis also leads to phenotype rescue, these data imply that the defects in endomembrane system and autophagy in *dga1Δ lro1Δ* cells are caused by the accumulation of an intermediate metabolite, with PA and DAG being the primary suspects.

Starvation invokes complex changes in the signaling network. To examine if the connection under investigation between lipids and the endomembrane system depends on starvation, we boosted glycerolipid synthesis in cells under nutrient-rich condition by supplementing the growth medium with oleic acid (OA). While wild-type cells were tolerant to OA, *dga1Δ lro1Δ* cells suffered disturbance in the endomembrane system (Additional file 1: Fig. S3A-B). The responses were overall similar to those under starvation, albeit with a more prominent redistribution of ER markers to the bulbs, leaving the regular ER network faintly visible (Additional file 1: Fig. S3A). Possibly because of this stronger ER response, the ER recovery brought by *opi1Δ* was only partial. As acyl-CoA synthetases are essential in the assimilation of exogenous fatty acids, OA-induced disruptions were completely prevented by *faa1Δ faa4Δ*. For *slc1Δ*, the recovery of organelle morphology was again partial. These data imply that with or without starvation, the same lipids are causing the disruptive effects.

### Block in TAG synthesis leads to concentration of DAG at the ER

**Fig. 5:**
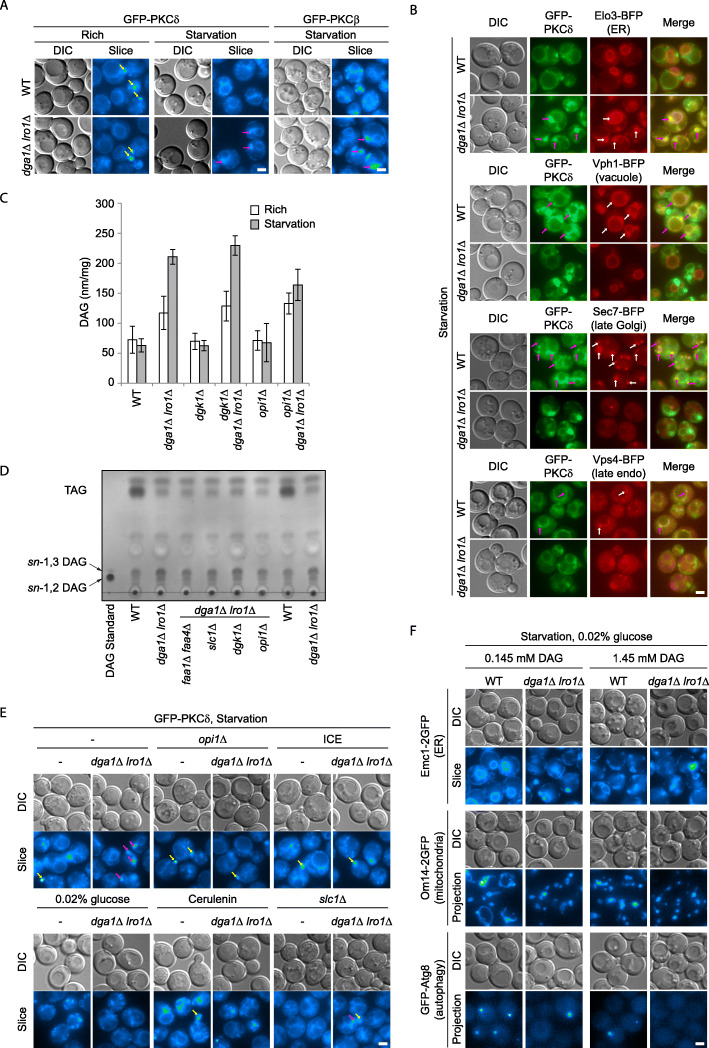
Accumulation of DAG at the ER in TAG production defective cells. **a** Starvation triggers intracellular DAG accumulation in *dga1Δ lro1Δ* cells. Cells of the indicated genotype expressing DAG probes (GFP-PKCδ and GFP-PKCβ) were grown to mid-log phase and then starved for 1 h. Images presented as in Fig. 1a. Yellow arrows, concentration of DAG at the buds in normal cells. Purple arrows, accumulation of DAG at intracellular bulbs. Scale bar, 2 μm. **b** Starvation triggers DAG accumulation at the ER in *dga1Δ lro1Δ* cells. Cells treated as in **a**, except that additional organelle markers (ER, vacuole, late Golgi, and late endosome) were co-expressed. Image presented as in Fig. 2a. Arrows, incidences of GFP-PKCδ colocalization with organelle markers. Scale bar, 2 μm. **c**, **d** Total cellular DAG. Cells of the indicated genotype were grown to mid-log phase and then starved for 1 h. Lipids were extracted and analyzed by mass spectrometry-assisted quantification (**c**) or thin layer chromatography (TLC) (**d**). **c** Error bar, standard deviation, *n* = 3. **d** Representative image from three independent repeats. **e** Manipulation of glycerolipid synthesis pathway alters DAG localization. Phospholipid production was upregulated by (1) knocking out *OPI1* or (2) supplying key reactants (inositol, choline, and ethanolamine/ICE). Precursor influx was constrained by (1) 100-fold reduction of glucose supply (0.02% glucose), (2) chemical inhibition of fatty acid synthase (cerulenin), or (3) elimination of a key lysoPA acyltransferase (*slc1Δ*). Cells were starved for 1 h. Images presented as in **a**. Scale bar, 2 μm. **f** Exogenous DAG induces endomembrane defects. 1,2-Dioctanoyl-sn-glycerol of the indicated concentrations was added to starvation medium containing 0.02% glucose. Cells were starved for 1 h. Images presented as in Fig. 1a. Scale bar, 2 μm

**Fig. 6:**
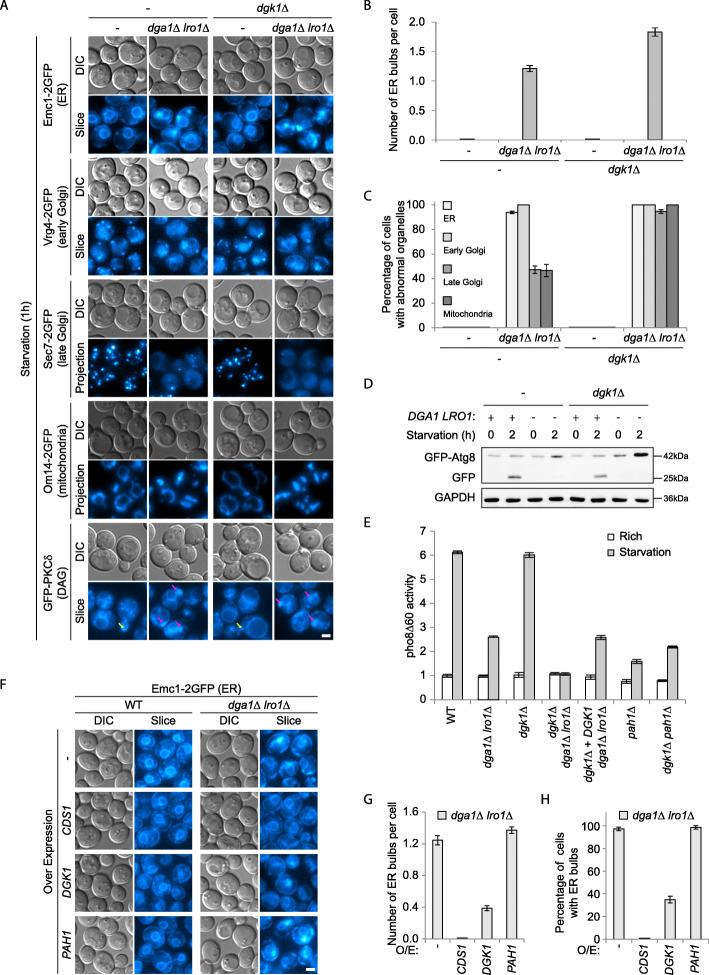
Excess DAG, but not PA, is the cause of endomembrane and autophagy defects in TAG production defective cells. **a–c** Knocking out *DGK1* aggravates endomembrane defects in *dga1Δ lro1Δ* cells. **a** Representative images presented as in Fig. 1a. Yellow arrows, concentration of DAG at the buds in normal cells. Purple arrows, ER bulbs. Scale bar, 2 μm. **b** Number of ER bulbs per cell. **c** Percentage of cells displaying abnormal organelle morphology (ER bulb formation, Golgi disappearance, mitochondrial fragmentation). Error bar, standard deviation, *n* = 3. **d**, **e** Knocking out *DGK1* aggravates autophagy defect in *dga1Δ lro1Δ* cells. Autophagic flux was measured by **d** proteolytic processing of GFP-Atg8, and **e** pho8Δ60 assay. Results presented as in Fig. 4e, f. **f**–**h** Overexpression of *DGK1* and *PAH1* produces opposite effects on ER bulb formation in *dga1Δ lro1Δ* cells. **f** Representative images presented as in Fig. 1a. Scale bar, 2 μm. **g** Number of ER bulbs per cell. **h** Percentage of cells displaying ER bulbs. Error bar, standard deviation, *n* = 3

We then examined the subcellular localization and total amount of DAG. In both wild-type and *dga1Δ lro1Δ* cells under growing condition, DAG was predominantly present at the following sites: nascent buds, vacuolar membrane, late endosomes, and late Golgi/early endosomes, as indicated by the signal of a fluorescent protein probe based on PKCδ C1 domain (GFP-PKCδ) (Fig. 5a, b) [38]. The signal on the buds was the brightest. No signal was discernable on the early Golgi (data not shown). Only some very faint signal of the probe was on the ER. Starvation did not change the overall distribution of DAG in wild-type cells other than reducing the number of nascent buds (Fig. 6a). In contrast, *dga1Δ lro1Δ* cells displayed a dramatic shift of the probe from the aforementioned sites to the ER (Fig. 5a, b). The signal of the probe was concentrated on the bulbous structures, colocalizing with ER proteins therein. We also examined the distribution of DAG with a second probe based on PKCβ C1 domain (GFP-PKCβ), which displayed similar relocation to ER bulbs in *dga1Δ lro1Δ* cells (Fig. 5a). The change in subcellular DAG distribution was accompanied by an approximately threefold increase in total DAG level, as revealed by lipidomic quantification (Fig. 5c) and thin layer chromatography (TLC) (Fig. 5d). Furthermore, the extent of DAG accumulation in *dga1Δ lro1Δ* cells could be partially mitigated by either increased consumption (*opi1Δ*) or decreased precursor supply (*faa1Δ faa4Δ*, or *slc1Δ*). Consistently, manipulations on intermediate consumption and precursor supply also completely (*opi1Δ*, ICE, 0.02% glucose, or *faa1Δ faa4Δ*) or partially (*slc1Δ*) eliminated the relocation of the DAG probe to the ER (Fig. 5e).

### Excess DAG, not PA, causes endomembrane defects in TAG production defective cells

Consistent with DAG accumulation being a candidate causal factor for the disruptions, we found that the addition of 1,2-dioctanoyl-sn-glycerol (a cell permeable DAG analog) led to ER bulb formation, mitochondria fragmentation, and autophagy inhibition in *dga1Δ lro1Δ* cells incubated in starvation medium containing 0.02% glucose (Fig. 5f).

The interconversion between PA and DAG is mainly mediated by two enzymes, Pah1 (PA phosphatase) and Dgk1 (DAG kinase) (Additional file 1: Fig. S1A) [31, 39, 40]. Deletion of *PAH1* also caused pleiotropic disturbances in the endomembrane system (Additional file 1: Fig. S4A). The nuclear ER/nuclear envelope was substantially enlarged in *pah1Δ* cells [41]. However, no bulbous structures were present. Both early Golgi and late Golgi/early endosome remained visible as multiple puncta, except that a fraction of early Golgi proteins also appeared on large sheet-like structures. Vacuoles became fragmented, as reported previously [42]. Instead of fragmenting into multiple spheres like in *dga1Δ lro1Δ* cells, mitochondria in *pah1Δ* cells became somewhat clustered, and the remaining tubular structures were shorter and less interconnected. Except for vacuolar fragmentation, all the changes described above were already present in *pah1Δ* cells under growing condition (Additional file 1: Fig. S4A). The changes became more severe as *pah1Δ* cells entered starvation. Note that autophagy was also inhibited in *pah1Δ* cells, yet *OPI1* knockout did not relieve the inhibition (Additional file 1: Fig. S4B). As the alterations of the endomembrane system in *pah1Δ* cells were drastically different from that in *dga1Δ lro1Δ* cells, the underlying causes of their autophagy inhibition are likely distinct. In de novo glycerolipid synthesis, PA-to-DAG conversion is positioned immediate upstream of DAG-to-TAG conversion [31]. Accordingly, eliminating enzymes in both steps (*pah1Δ lro1Δ P*_*GAL*_*-DGA1* in glucose containing media) produced a phenotype mimicking that of *pah1Δ* (Additional file 1: Fig. S4C).

Deletion of *DGK1* alone did not generate any obvious phenotype. On the other hand, deletion of *DGK1* in the *dga1Δ lro1Δ* background exaggerated the morphological changes, with ER bulb formation, Golgi disappearance, and mitochondria fragmentation all being more severe than *dga1Δ lro1Δ* alone (Fig. 6a–c). These defects also showed up sooner in the triple knockout cells (data not shown). The levels of DAG in *dgk1Δ dga1Δ lro1Δ* cells were slightly higher than those in *dga1Δ lro1Δ* cells, although the differences were not statistically significant (Fig. 5c). Autophagic flux in *dgk1Δ dga1Δ lro1Δ* cells was lower than that in *dga1Δ lro1Δ* cells, an effect that could be complemented by re-expression of *DGK1* (Fig. 6d, e). In contrast, knocking out *DGK1* slightly enhanced autophagy in *pah1Δ* cells (Fig. 6e). These data support a hypothesis that defects in *dga1Δ lro1Δ* cells and *pah1Δ* cells are each caused by the accumulation of DAG and PA, which *dgk1Δ* aggravates and alleviates, respectively.

Consistent with excess DAG being the endomembrane disruptor, overexpression of *DGK1* led to partial restoration of ER morphology, whereas overexpression of *PAH1* led to slight enhancement in ER bulb formation in *dga1Δ lro1Δ* cells (Fig. 6f–h). A more robust restoration was observed with CDS1 overexpression, which diverts glycerolipid synthesis pathway towards phospholipids.

### ER pool of DAG is distinct from those generated at the Golgi in sphingolipid synthesis

Considering the drastic effect of DAG on the maintenance of the Golgi, we explored whether the Golgi pool of DAG may contribute to the endomembrane disruption in cells. This pool of DAG is generated from sphingolipid biosynthesis [43, 44] and has been proposed to promote vesicular trafficking out of the Golgi [45].

The pattern of ER marker distribution in *dga1Δ lro1Δ* cells was unaffected by the depletion of Aur1, the first enzyme in this set of reactions (Additional file 1: Fig. S5A) [46]. Addition of an Aur1 inhibitor, aureobasidin A (AbA), up to lethal doses to *dga1Δ lro1Δ* cells did not affect their respective ER morphologies (Additional file 1: Fig. S5B). Myriocin, an inhibitor of serine-palmitoyl transferase (SPT) responsible for the first step in sphingolipid synthesis, did not prevent ER bulb formation either (Additional file 1: Fig. S5B). These data indicate that DAG generated from sphingolipid metabolism does not have a substantial role in the disruption of endomembrane system in *dga1Δ lro1Δ* cells. We suspect that the amount of DAG from this source is insignificant or that there is a transportation barrier preventing it from joining the ER pool.

### Effects of intracellular DAG do not depend on Pkc1 or the unfolded protein response

One potential downstream target of DAG is C1 domain containing proteins, including members of the protein kinase C (PKC) family [47, 48]. Pkc1 is both the only PKC and the only C1 domain containing protein in yeast. Whether Pkc1 is regulated by DAG has been controversial in existing literature. We found that the subcellular localization of Pkc1 differed from that of GFP-PKCδ. As reported [49], Pkc1 was primarily concentrated at the nascent buds and bud necks (Additional file 1: Fig. S6A). The pattern of Pkc1 localization in *dga1Δ lro1Δ* cells was comparable to that in wild-type cells under both growing and starvation conditions (Additional file 1: Fig. S6A). In addition, we deleted genes mediating Pkc1 downstream signaling (*BCK1*, *SLT2*, *MKK1*, and *MKK2*) in *dga1Δ lro1Δ* cells and observed no discernable effect on the degree of ER bulb formation and mitochondria fragmentation (Additional file 1: Fig. S6B) [50]. We also investigated the potential involvement of the unfolded protein response (UPR). Treating wild-type cells with dithiothreitol (DTT) or tunicamycin triggered the UPR, as evidenced by the shift in Ire1 subcellular distribution from diffuse to punctate (Additional file 1: Fig. S6C) [51]. The same change also occurred in *dga1Δ lro1Δ* cells upon starvation (Additional file 1: Fig. S6C), indicating the presence of ER stress. However, DTT and tunicamycin were unable to induce ER bulb formation (Additional file 1: Fig. S6D). ER bulbs in cells lacking Dga1 and Lro1 were not reduced by knocking out *IRE1* (Additional file 1: Fig. S6E), indicating that the UPR pathway is not essential for their formation. These data suggest that the effect of intracellular DAG is independent of Pkc1 signaling and the UPR and is potentially mediated by novel factors without typical C1 domains.

## Discussion

This work began as an exploration on the connection between TAG biosynthesis and autophagy, and ended up with the identification of ER-accumulated DAG as a key molecule, the excess of which disrupts the endomembrane system (Additional file 1: Fig. S7). Starvation, like many other stress conditions, induces TAG synthesis. Under this condition, blocking the conversion of DAG to TAG causes drastic accumulation of DAG, accompanied by its redistribution from the vacuole membrane to the ER (Fig. 5). The accumulation of DAG at the ER, but not Golgi or mitochondria, also implies that the ER is the primary site of the regulatory action of DAG. This is further underscored by the fact that DAG generated at the Golgi has no significant role in the observed organelle changes (Additional file 1: Fig. S5). Disturbance of ER function may contribute to the change in mitochondrial morphology, as the ER is intimately involved in mitochondrial fission [52, 53]. The resulting relocation of Golgi marker proteins to the ER indicates that the balance of ER-Golgi trafficking pathways is severely disturbed, potentially compromising the functions of both organelles, leading to impaired Atg protein recruitment and defective autophagosome biogenesis (Figs. 1 and 2).

Our assignment of DAG as the root cause is supported by data showing that all manipulations that reduce DAG levels, either by decreasing the supply of its precursors or by increasing its consumption by phospholipid synthesis, restored the morphology of the endomembrane system and autophagy (Figs. 3 and 4). Conversely, boosting DAG levels by blocking its conversion to PA in *dga1Δ lro1Δ* cells results in even stronger disruptions to the organelles (Fig. 6). Furthermore, the effects of DAG accumulation are profoundly different from that of PA accumulation, even though both can inhibit autophagy. DAG causes the formation of ER bulbs and the regression of Golgi (Figs. 1 and 2). In contrast, PA induces the expansion of the nuclear ER and the fragmentation of the vacuole (Additional file 1: Fig. S4). Lastly, the assignment of DAG as the causal factor is fully consistent with data independently published by other colleagues [34]. Velazquez et al. previously hypothesized that autophagy inhibition in *dga1Δ lro1Δ* cells originates from the accumulation of FA. Indeed, we found that inhibition of fatty acid synthase by cerulenin rescued autophagy. However, we also found that cerulenin effectively prevented ER accumulation of DAG (Fig. 5). Moreover, we show that elimination of fatty acyl-CoA synthetases (*faa1Δ faa4Δ*), which decreases DAG by blocking FA utilization (Additional file 1: Fig. S1A), led to recovery of organelle morphology and autophagy (Figs. 3 and 4). These data indicate that FA per se is not the autophagy-inhibitory molecule and that the rescuing effect of cerulenin is instead mediated by changes in DAG.

DAG is both an intermediate metabolite and a signaling molecule. In the second role, it is best known as a second messenger generated from hydrolysis of phospholipids at the PM [48, 54]. DAG binding proteins, including C1 domain containing proteins, then mediate the downstream signaling [47]. In addition to the PM, DAG is also known to function at intracellular sites. In mammalian cells, DAG is mainly present at the Golgi, ER, and nuclear envelop (NE) [55]. At the Golgi, DAG promotes the formation and/or fission of coated vesicles, in which protein kinase D (PKD) acts as a downstream target [56–59]. Furthermore, Golgi DAG participates in RAS signaling and the regulation of ER morphology [60, 61]. Upon bacterial invasion, DAG labels bacterial containing phagosomes and promotes antibacterial autophagy through activation of PKCδ [62]. In sea urchin oocytes, manipulation of DAG regulates the conversion between ER sheets and tubules [63]. Evidence of intracellular functions of DAG has also been documented in yeast, including vesicular trafficking from the Golgi and homotypic vacuole fusion, albeit direct protein targets of DAG are unknown [45, 64, 65]. Collectively, these studies demonstrate the critical need of DAG in a variety of cellular functions at both the PM and intracellular membranes.

Conversely, an oversupply of DAG may produce detrimental effects, which is the topic of the present study. Notably, DAG accumulation triggers cell death in both fission yeast and budding yeast [8, 66]. Although the issue of subcellular localization was not directly examined, both studies employed double mutants of *DGA1* and *LRO1* or their homologs, similar to our study, indicating that ER may also be the primary site of DAG action in cell death pathways. ER morphological abnormalities have also been observed in *dga1Δ lro1Δ are1Δ are2Δ* cells [34, 67, 68], which are defective in the synthesis of both TAG and sterol esters. The precise outcomes of ER morphology in *dga1Δ lro1Δ are1Δ are2Δ* cells appear to vary, possibly reflecting differences in experimental conditions as well as contributions from other lipid species. Nevertheless, all studies demonstrate a strong link between storage lipid metabolism and ER homeostasis. In particular, ER membrane aggregation in post-diauxic *dga1Δ lro1Δ are1Δ are2Δ* cells was exaggerated by *dgk1Δ* [67], again consistent with DAG being the causal factor in this particular type of organelle alteration. Excess DAG’s disruptive role in ER is potentially conserved in animals. In adipose tissue-specific DGAT1 knockout mice, high-fat diet led to significant upregulation of unfolded protein response [69]. In human adipose tissue samples, there is also a negative correlation between the expression of DGAT1 and ER stress genes [70]. In most aforementioned studies, excess DAG is generated at the ER during the biosynthesis of glycerolipids. As a result, this pool of DAG is controlled by the balance of lipid metabolism and may act chronically, producing a long-term health risk.

At present, it is not clear how excess DAG triggers the disruption of the ER and Golgi. It is possible that the effects are mediated by one or more novel downstream proteins or by direct modification of the biophysical properties of cellular membranes [71, 72]. DAG is a cone-shaped glycerolipid with a small head, the hydroxyl group. Thus, its presence in membrane favors negative spontaneous curvature. Furthermore, it is capable of fast flip-flop across lipid bilayers, which over time may negate existing membrane curvature. Both vesicle budding and fusion involve stage-specific and location-specific alterations in membrane curvature [72]. From this perspective, the concentration of DAG in membrane needs to be strictly controlled to ensure normal vesicular trafficking. At high DAG molar ratios, phase separation occurs and lens-like domain consisting primarily of DAG may form within lipid bilayers [71, 73]. The concentration of DAG in total yeast lipid is approximately 4% [74]. If the concentration of DAG in our samples is comparable with those reported, a threefold increase would put it dangerously close to the threshold of phase separation in theoretical simulations. The local concentration in ER can be even higher. The lens formation is analogous to the behavior of TAG in a proposed model of lipid droplet formation [1]. We checked ER bulb formation in two mutants of lipid droplet homeostasis, *fld1Δ* and *ice2Δ*, but saw no obvious impact (Additional file 1: Fig. S6E). This does not rule out the possibility that other protein factors are actively involved in ER bulb formation, however. Further research is needed to elucidate the underlying mechanism.

## Conclusions

In summary, this work identifies DAG as the critical lipid molecule responsible for autophagy inhibition under condition of defective TAG synthesis. Our data further implicate the disruption in ER and Golgi homeostasis as a major driving force behind observed autophagy impairments, which is in turn caused by the excess accumulation of DAG at the ER.

## Methods

### Plasmids and strains

Strains and plasmids constructed in this study are listed in Additional file 2: Tables S1 & S2. Plasmids for additional organelle markers were described previously [75, 76]. Restriction sites and PCR primers utilized in plasmid construction are listed in Additional file 2: Table S3. Linearization sites and loci for genome integration of plasmids are also listed in Additional file 2: Table S2. DNA sequences of the primers used in plasmid construction are listed in Additional file 2: Table S3. Gene knockouts were performed using common homologous recombination-based technique [77, 78].

### Culturing of yeast cells

Yeast cells in liquid culture were incubated at 30 °C with shaking. Media recipes for YPD, SD-N, and SMD amino acid dropouts were described previously [76, 79]. Unless otherwise noted, YPD was used to grow cells to mid-log phase and SD-N was used for nitrogen starvation. When indicated, the following chemicals were added to culture media at the designated final concentrations: cerulenin (10 μg/ml), 1,2-dioctanoyl-sn-glycerol (0.145 mM, 1.45 mM), ICE (inositol, 0.05 mM; choline, 1 mM; ethanolamine, 1 mM), oleic acid (0.5 mM), myriocin (10 μg/ml), and aureobasidin A (AbA, 1 μg/ml).

For experiments involving GAL promoter-driven constructs, yeast strains were first re-streaked on YPGal plates. Strains were then inoculated in YPD and grown overnight to mid-log phase before shifting to SD-N medium. For experiments involving *P*_*GAL*_*-AUR1*, the expression of Aur1-2GFP was monitored by fluorescent microscopy once per hour. Cells were shifted to SD-N medium once the GFP signal became undetectable.

### TEM

Yeast cells were first fixed in PHEM buffer (20 mM PIPES, 50 mM HEPES, 20 mM EGTA, 4 mM MgCl_2_, adjust the PH to 6.9) containing 2% formaldehyde and 0.2% glutaraldehyde for 3 h at room temperature and washed three times in PHEM buffer. Cells were then incubated in DPBS (Dulbecco’s phosphate-buffered saline) buffer containing 1.4 mM DAB and 0.5 mM H_2_O_2_ and kept in darkness at room temperature for 20 min, after which cells were washed three times in DPBS. Afterwards, cells were fixed a second time in 2% KMnO_4_ and kept at 4 °C for 3 h. Cells were then washed with water several times till supernatant became colorless.

Subsequently, samples were dehydrated using graded series of ethanol (30%, 50%, 70%, 80%, 90%, then 100% three times), infiltrated with LR White resin (EMS) (33%, 66%, then 100% three times, 12 h per step at room temperature), and polymerized at 60 °C for 48 h. Samples were sectioned to 70 nm thin. Images were acquired on a transmission electron microscope.

### Total lipid extraction

Total lipids were extracted using a modified protocol of Zhu et al. [80]. Yeast cells were pelleted and freeze-dried. Samples were then suspended in 500 μl chloroform-methanol (2:1, v/v) with glass beads and underwent 15 min of mechanical shearing on a bead-mill. Lysates were centrifuged at 10,000*g* for 5 min, and the organic phase was moved to new tubes for collection. The chloroform-methanol extraction procedure was repeated five times. The organic phase collections were merged and evaporated at 60 °C.

### Lipid analysis by TLC

TLC analysis of lipids was performed essentially as described in [81]. Lipid extracts were dissolved in chloroform and spotted on activated silica gel plates. Neutral lipids were separated using hexane-diethyl ether-acetic acid (70:30:1 v/v/v) as the mobile phase. Plates were stained by a 0.03% Coomassie Brillant Blue R-250 solution containing 20% methanol for 15 min and de-stained by 20% methanol for 10 min [82].

### Lipid analysis by LC/MS

For DAG, total lipids were extracted in the presence of internal standards (C19:0/C19:0 DAG) and analyzed on a TSQ Vantage mass spectrometer equipped with an electrospray ion source (ThermoFisher Scientific) and coupled to a UHPLC system with an ACQUITY® UPLC HILIC column (100 × 2.1 mm, 1.7 μm, Waters). The flow rate was set at 0.2 ml/min, and the following mobile phase gradient was employed: 0 min 99% A + 1% B, 10 min 80% A + 20% B, 11 min 2% A + 98% B, and then held for 2 min, with A being ACN/H_2_O (95:5, v/v, 10 mM ammonium acetate) and B being ACN/H_2_O (50:50, v/v, 10 mM ammonium acetate). Molecular species of DAG were analyzed following one-step derivatization with *N*,*N*-dimethylglycine as previously described [83].

### Others

Fluorescence microscopy, pho8Δ60 assay, and GFP-Atg8 processing assay were performed as described previously [75, 76, 79]. For quantification of microscopy images, the experiments were repeated at least 3 times, and a total of at least 150 cells were analyzed for each sample.

## Supplementary information

**Additional file 1: Figure S1.** TAG utilization is not essential for autophagy. **Figure S2.** Fluorescent microscopy of additional organelle markers. **Figure S3.** Intermediate accumulation is responsible for endomembrane defects in TAG production defective cells under growing condition upon oleic acid addition. **Figure S4.** PA accumulation leads to distinct alterations in the endomembrane system. **Figure S5.** ER bulb formation in TAG production defective cells does not rely on Golgi produced DAG. **Figure S6.** Endomembrane defects in TAG production defective cells do not rely on Pkc1 signaling or UPR. **Figure S7.** Schematic depiction for the role of DAG in the endomembrane system.

**Additional file 2: Table S1.** Strains used in this study. **Table S2.** Plasmids constructed in this study. **Table S3.** DNA sequences of PCR primers.

**Additional file 3.** Raw data in excel file for all column graphs in figures and additional figures.

## Data Availability

Raw data for column graphs are presented in Additional file 3: Raw Data.
