# Supplementary material for: Excess diacylglycerol at the endoplasmic reticulum disrupts endomembrane homeostasis and autophagy
Source: BMC Biol. 2020 Aug 28;18:107. doi: 10.1186/s12915-020-00837-w (PMC7453538; doi:10.1186/s12915-020-00837-w)
Supplement: Supplementary file 1 — Additional file 1: Figure S1. TAG utilization is not essential for autophagy. Figure S2. Fluorescent microscopy of additional organelle markers. Figure S3. Intermediate accumulation is responsible for endomembrane defects in TAG production defective cells under growing condition upon oleic acid addition. Figure S4. PA accumulation leads to distinct alterations in the endomembrane system. Figure S5. ER bulb formation in TAG production defective cells does not rely on Golgi produced DAG. Figure S6. Endomembrane defects in TAG production defective cells do not rely on Pkc1 signaling or UPR. Figure S7. Schematic depiction for the role of DAG in the endomembrane system. [file 12915_2020_837_MOESM1_ESM.pdf]

Fig. S1

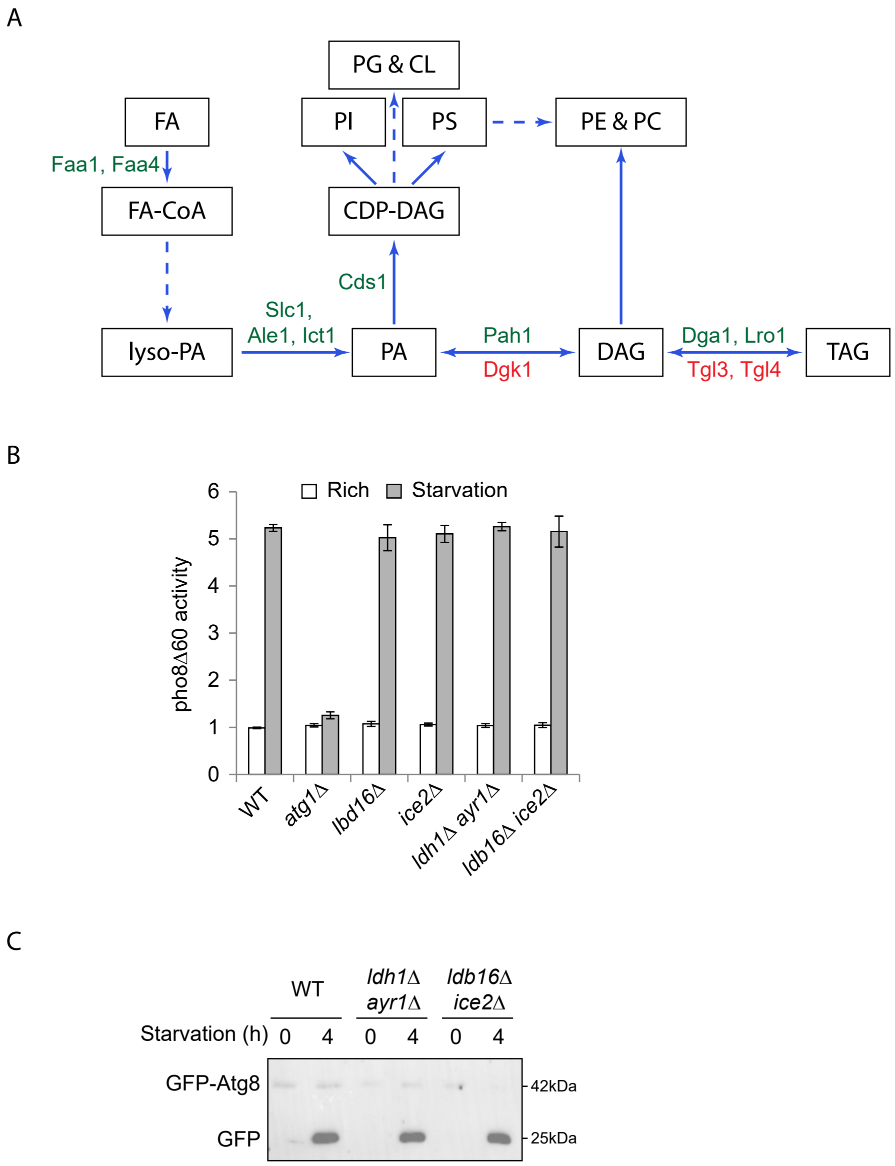
A
PG & CL
FA
PI
PS
PE & PC
Faa1, Faa4
FA-CoA
CDP-DAG
Cds1
Slc1,
Ale1, Ict1
lyso-PA
PA
Pah1
Dgk1
DAG
Dga1, Lro1
Tgl3, Tgl4
TAG
B
Rich
Starvation
pho8Δ60 activity
6
5
4
3
2
1
0
WT
atg1Δ
lbd16Δ
ice2Δ
ldh1Δ ayr1Δ
ldb16Δ ice2Δ
C
WT
ldh1Δ ayr1Δ
ldb16Δ ice2Δ
Starvation (h)
0
4
0
4
0
4
GFP-Atg8
GFP
42kDa
25kDa

Figure S1. TAG utilization is not essential for autophagy.

(A) Schematic summary of yeast glycerolipid biosynthesis pathway. Dashed lines represent multiple reactions. For key reactions relevant to this study, the names of the enzymes are listed. A double headed arrows represents a pair of reactions interconverting the two molecules; enzymes in red fonts underneath the double headed arrow catalyze the conversion from the right to the left.

(B, C) Autophagy under nitrogen starvation does not depend on several proteins hypothesized to participate in TAG utilization. Autophagic flux was assessed using pho8Δ60 assay **(B)** and GFP-Atg8 processing assay **(C)**. Results presented as in Fig. 4F and 4E.

Fig. S2

A

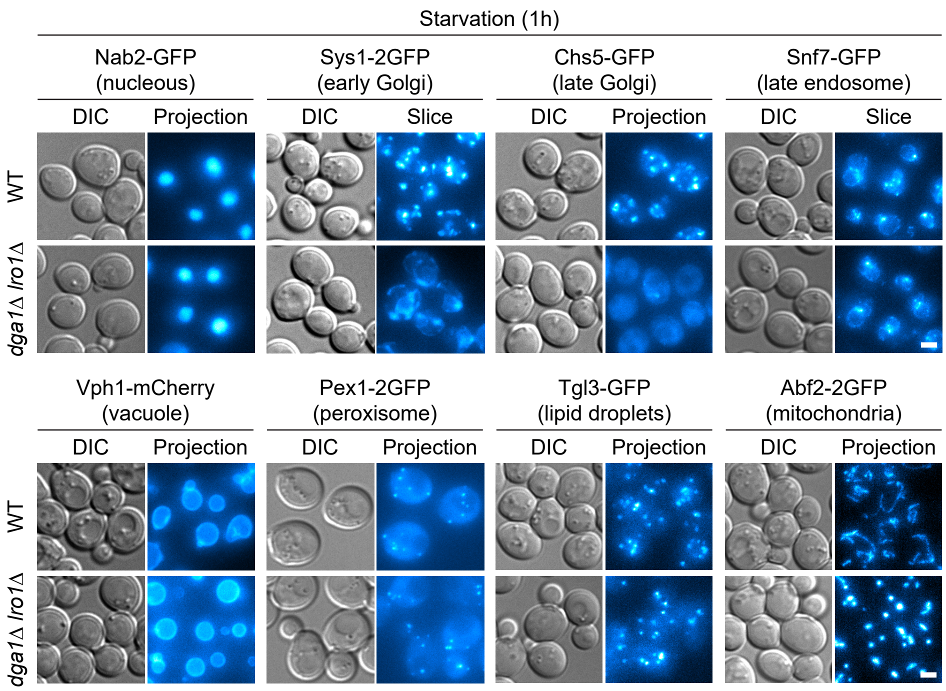

Starvation (1h)
Nab2-GFP
(nucleous)
Sys1-2GFP
(early Golgi)
Chs5-GFP
(late Golgi)
Snf7-GFP
(late endosome)
DIC
Projection
DIC
Slice
DIC
Projection
DIC
Slice
WT
dga1Δ lro1Δ
Vph1-mCherry
(vacuole)
Pex1-2GFP
(peroxisome)
Tgl3-GFP
(lipid droplets)
Abf2-2GFP
(mitochondria)
DIC
Projection
DIC
Projection
DIC
Projection
DIC
Projection
WT
dga1Δ lro1Δ

Figure S2. Fluorescent microscopy of additional organelle markers.

Cells were starved for 1 h. Image presented as in Fig. 1A. Scale bar, 2 um.

A

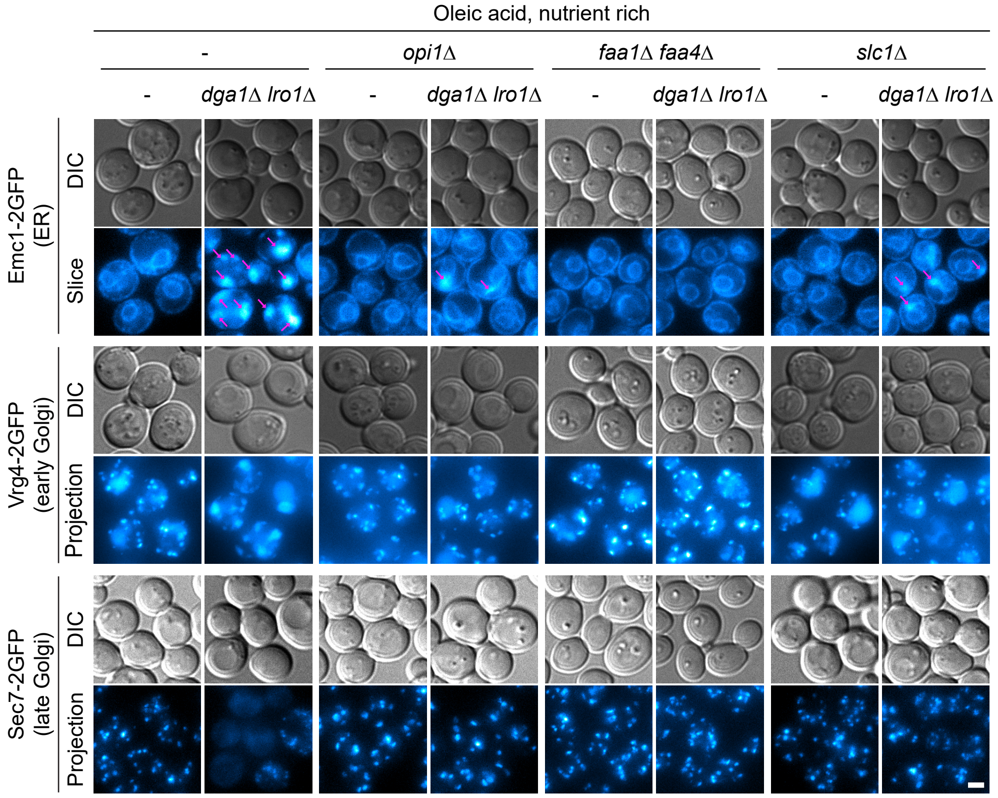
Oleic acid, nutrient rich
-
opi1Δ
faa1Δ faa4Δ
slc1Δ
-
dga1Δ lro1Δ
Emc1-2GFP (ER)
Vrg4-2GFP (early Golgi)
Sec7-2GFP (late Golgi)
DIC
Slice
Projection

B

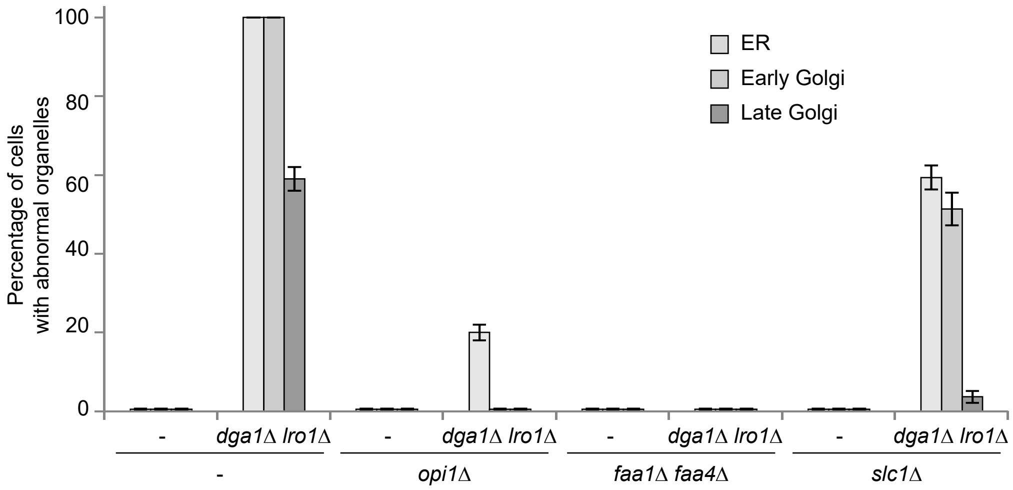
Percentage of cells with abnormal organelles
100
80
60
40
20
0
ER
Early Golgi
Late Golgi
-
dga1Δ lro1Δ
opi1Δ
faa1Δ faa4Δ
slc1Δ

Figure S3. Intermediate accumulation is responsible for endomembrane defects in TAG production defective cells under growing condition upon oleic acid addition.

Cells of the indicated genotypes were grown to mid-log phase and supplemented with 0.5 mM oleic acid for 1 h. Phospholipid production was upregulated by knocking out *OPI1*. Precursor influx was constrained by (1) elimination of major fatty acyl-CoA synthetases (*faa1Δ faa4Δ*), or (2) elimination of a key lysoPA acyltransferase (*slc1Δ*).

(A) Representative microscopy images presented as in Fig. 1A. Scale bar, 2 um.

(B) Quantification of cells displaying organelle defects. Error bar, standard deviation, n=3.

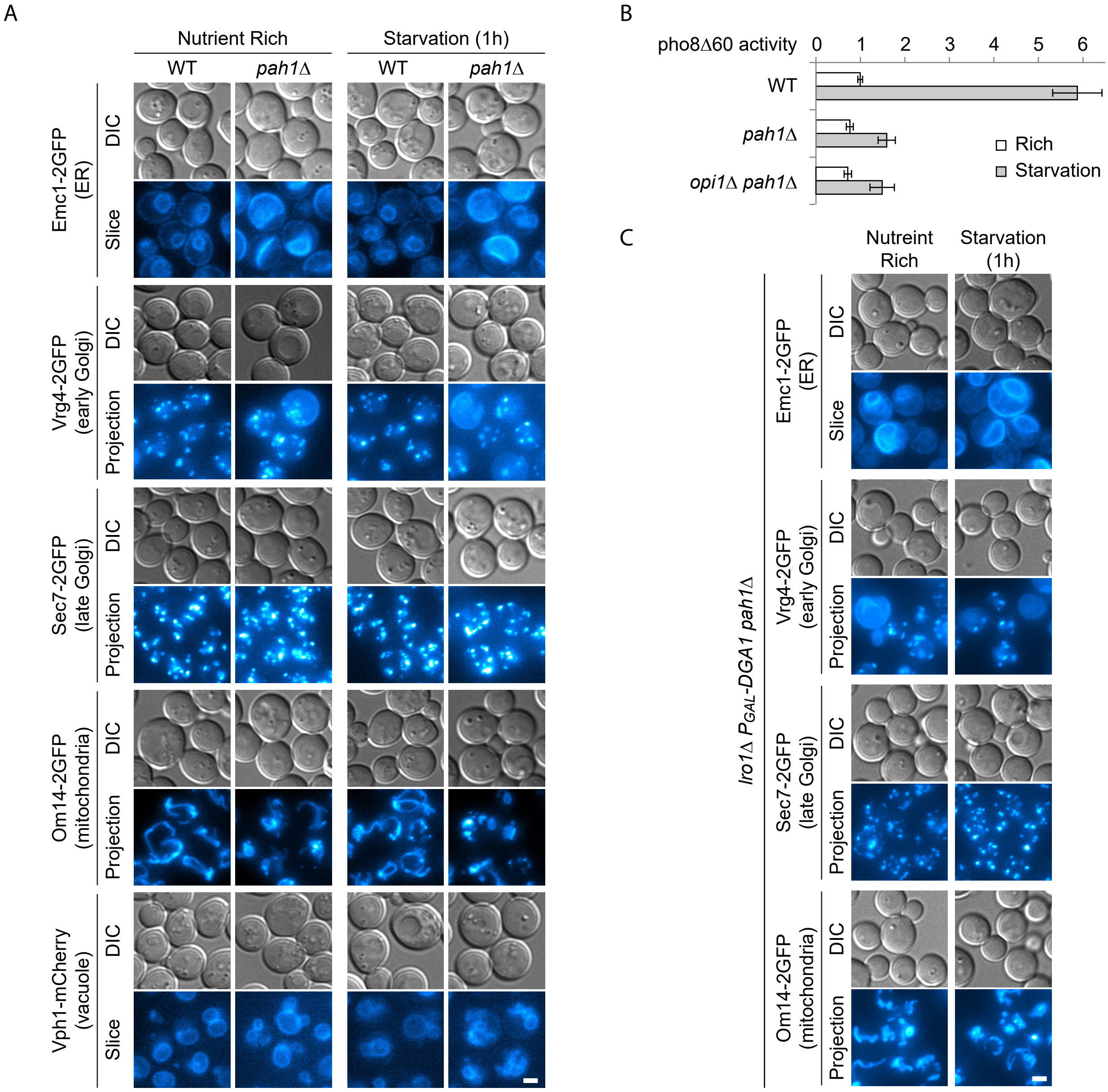
A
Nutrient Rich
Starvation (1h)
WT
pah1Δ
WT
pah1Δ
Emc1-2GFP (ER)
DIC
Slice
Vrg4-2GFP (early Golgi)
DIC
Projection
Sec7-2GFP (late Golgi)
DIC
Projection
Om14-2GFP (mitochondria)
DIC
Projection
Vph1-mCherry (vacuole)
DIC
Slice
B
pho8Δ60 activity
0
1
2
3
4
5
6
WT
pah1Δ
opi1Δ pah1Δ
Rich
Starvation
C
Nutreint Rich
Starvation (1h)
lro1Δ P$_{GAL}$-DGA1 pah1Δ
Emc1-2GFP (ER)
DIC
Slice
Vrg4-2GFP (early Golgi)
DIC
Projection
Sec7-2GFP (late Golgi)
DIC
Projection
Om14-2GFP (mitochondria)
DIC
Projection

Figure S4. PA accumulation leads to distinct alterations in the endomembrane system.

(A) PA accumulation alters the morphology of nuclear ER, mitochondria, and vacuoles. Images presented as in Fig. 1A. Scale bar, 2 um.

(B) Upregulation of phospholipid production by *opi1Δ* does not alleviate the autophagy defect in *pah1Δ* cells. Autophagic flux measured by pho8Δ60 assay. Error bar, standard deviation, n=3.

(C) Blocking both PA → DAG and DAG → TAG conversions mimics that of blocking PA → DAG conversion. In strains tested, *LRO1* and *PAH1* were knocked out, and *DGA1* was under the glucose-repressible *GAL* promoter. Images presented as in Fig. 1A. Scale bar, 2 um.

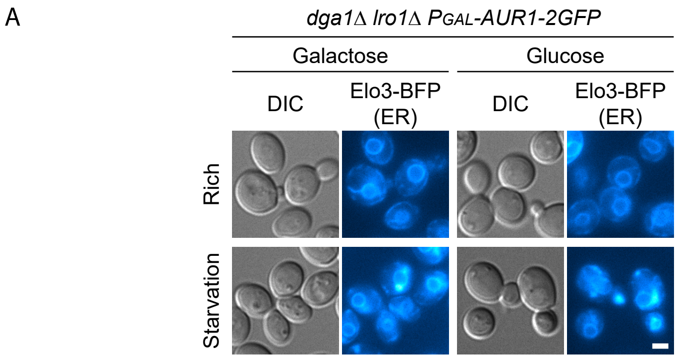
A
dga1Δ lro1Δ PGAL-AUR1-2GFP
Galactose
Glucose
DIC
Elo3-BFP (ER)
DIC
Elo3-BFP (ER)
Rich
Starvation

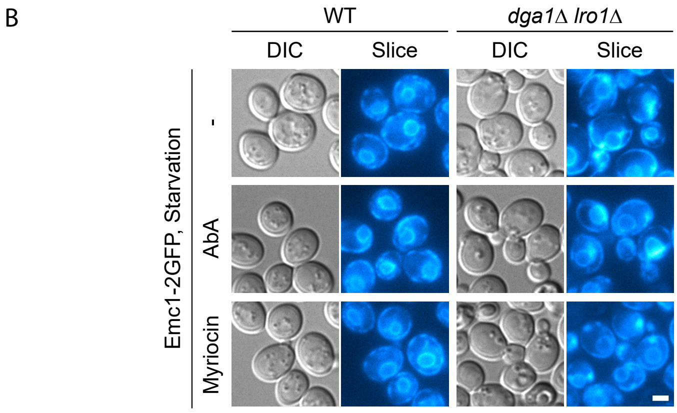
B
WT
dga1Δ lro1Δ
DIC
Slice
DIC
Slice
Emc1-2GFP, Starvation
-
AbA
Myriocin

Figure S5. ER bulb formation in TAG production defective cells does not rely on Golgi produced DAG.

(A) Depletion of IPC synthase (Aur1) does not prevent ER bulb formation. IPC synthase was depleted via transcriptional repression of P_{GAL}-*AUR1*. Cells were starved for 1 h. Images presented as in Fig. 1A. Scale bar, 2 um.

(B) Inhibition of serine palmitoyl transferase (myriocin) or IPC synthase (aureobasidin A, AbA) does not prevent ER bulb formation. Cells were starved for 1 h in the presence or absence of the inhibitors. Images presented as in Fig. 1A. Scale bar, 2 um.

Fig. S6

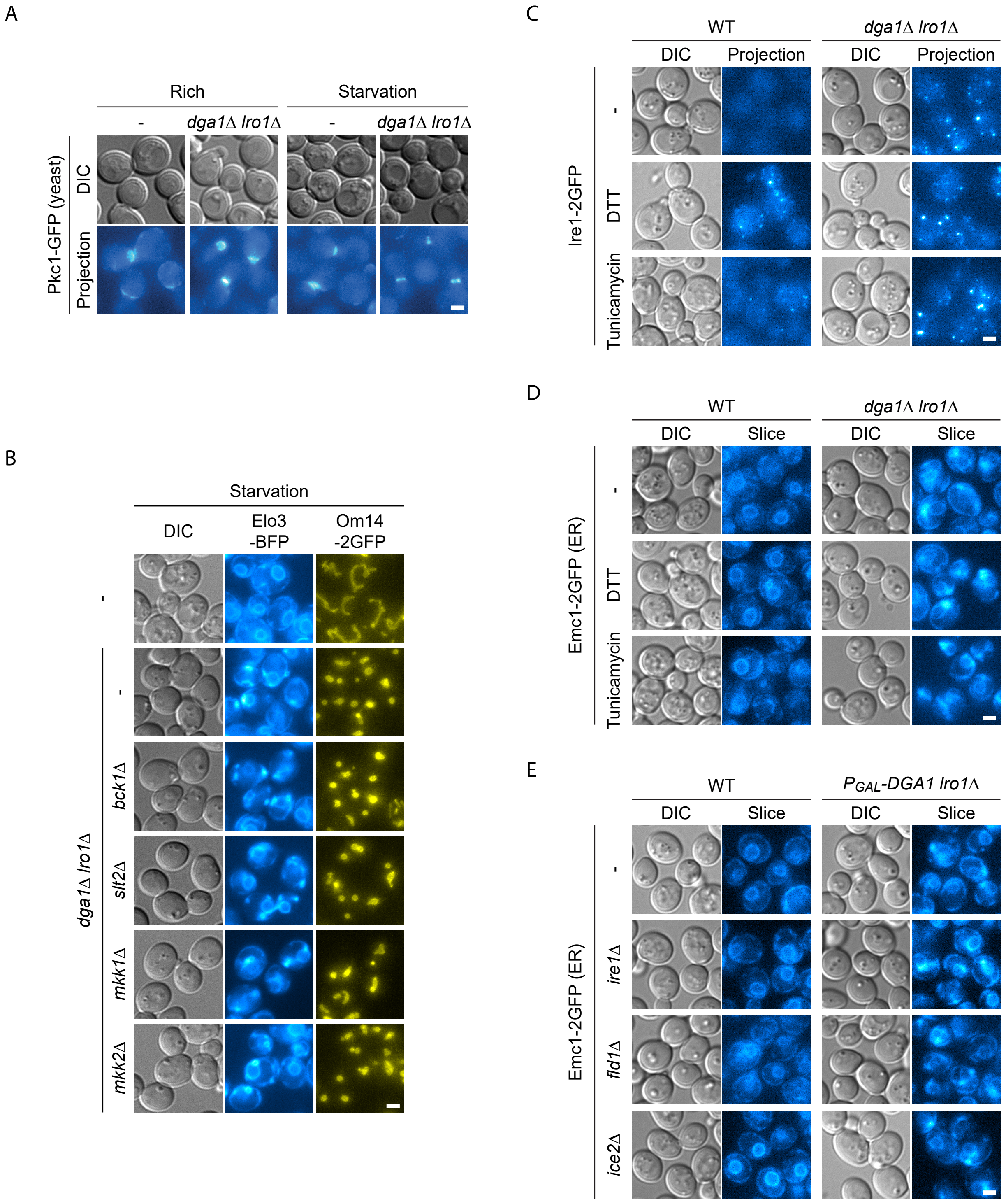
A
Rich
Starvation
-
dga1Δ lro1Δ
-
dga1Δ lro1Δ
Pkc1-GFP (yeast)
DIC
Projection
B
Starvation
DIC
Elo3 -BFP
Om14 -2GFP
-
dga1Δ lro1Δ
-
bck1Δ
slt2Δ
mkk1Δ
mkk2Δ
C
WT
dga1Δ lro1Δ
DIC
Projection
DIC
Projection
Ire1-2GFP
-
DTT
Tunicamycin
D
WT
dga1Δ lro1Δ
DIC
Slice
DIC
Slice
Emc1-2GFP (ER)
-
DTT
Tunicamycin
E
WT
PGAL-DGA1 lro1Δ
DIC
Slice
DIC
Slice
Emc1-2GFP (ER)
-
ire1Δ
fld1Δ
ice2Δ

Figure S6. Endomembrane defects in TAG production defective cells do not rely on Pkc1 signaling or UPR.

(A) Subcellular localization of yeast Pkc1 is DAG insensitive. Images presented as in Fig. 1A. Scale bar, 2 um.

(B) Endomembrane disruption by DAG is independent of Pkc1 signaling. Morphology of ER and mitochondria was observed in cells carrying knockouts of key genes in the MAPK pathway downstream of Pkc1. Representative image projections from individual channels are shown. Scale bar, 2 um.

(C) Upregulation of UPR in starved *dga1Δ lro1Δ* cells. The subcellular localization of Ire1, a key UPR pathway protein, was examined by fluorescent microscopy. Cells were first grown to mid-log phase, then shifted to starvation medium for 1 h. As indicated, DTT and tunicamyin were added at the time of media shift. Images presented as in Fig. 1A. Scale bar, 2 um.

(D) UPR alone does not lead to ER bulb formation. Cells treated and image presented as in (C), except for the fluorescent protein expressed.

(E) ER bulb formation does not require Ire1, Fld1, or Ice2. Cells treated and images presented as in Fig. 1A. Scale bar, 2 um.

Fig. S7

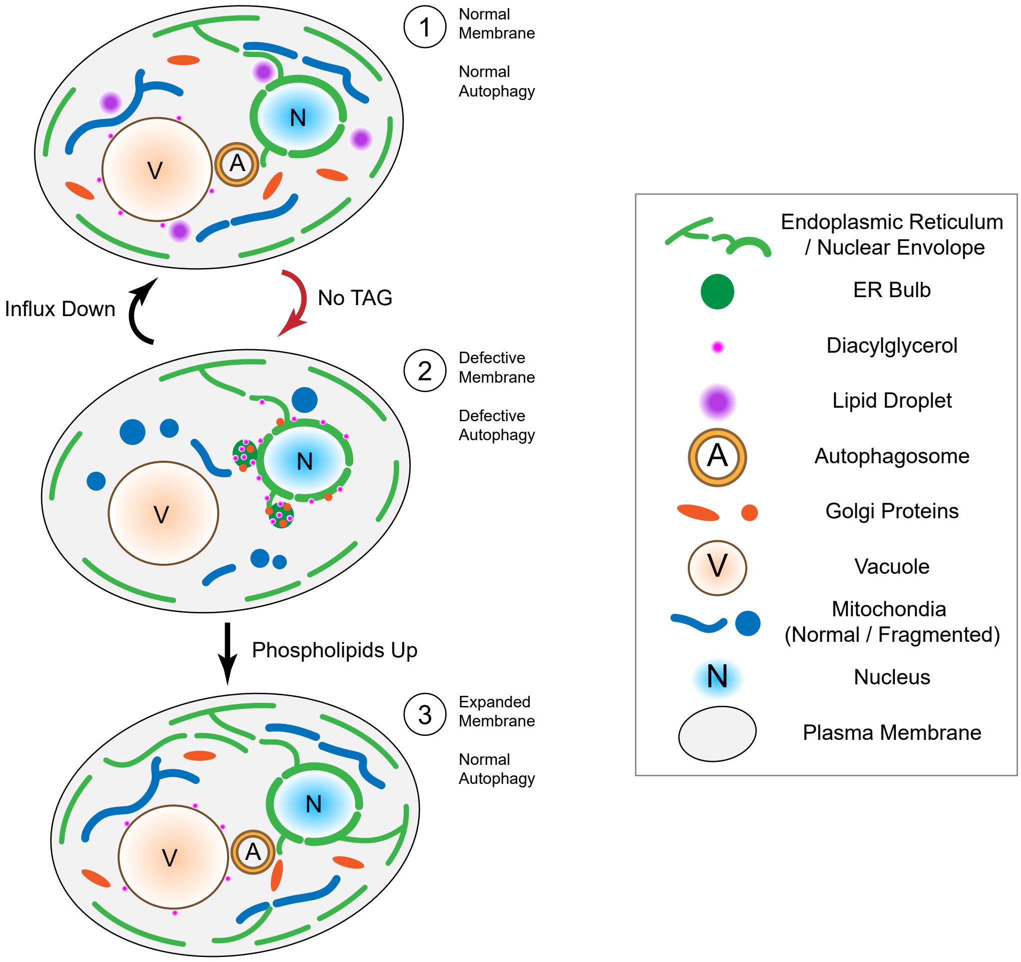

1
Normal Membrane
Normal Autophagy
Influx Down
No TAG
2
Defective Membrane
Defective Autophagy
Phospholipids Up
3
Expanded Membrane
Normal Autophagy
N
V
A
Endoplasmic Reticulum / Nuclear Envelope
ER Bulb
Diacylglycerol
Lipid Droplet
Autophagosome
Golgi Proteins
Vacuole
Mitochondia (Normal / Fragmented)
Nucleus
Plasma Membrane

Figure S7. Schematic depiction for the role of DAG in the endomembrane system.

(1) In wild-type cells under stress conditions (including nitrogen starvation), both autophagy and TAG synthesis are induced. TAG is stored in lipid droplets. DAG is mainly present on the vacuolar membrane.

(2) When TAG production is blocked (no TAG, i.e. *dga1Δ lro1Δ*), DAG accumulates in the ER, causing the formation of ER bulbs, disappearance of Golgi apparatus, fragmentation of mitochondria, and inhibition of autophagy. Golgi proteins either go back to the ER or become diffuse in the cytosol (not shown). Lipid droplet formation is compromised. *dga1Δ lro1Δ* cells can return to normal if lipid precursor supply is constrained (influx down).

(3) Excess DAG in *dga1Δ lro1Δ* cells can also be consumed by upregulation of phospholipid synthesis (phospholipids up, e.g. ICE, *opi1Δ*, Cds1 O/E). Other than an expanded peripheral ER, the endomembrane system returns to normal. Autophagy is restored with either “influx down” or “phospholipids up”.
