# Supplementary material for: Excess diacylglycerol at the endoplasmic reticulum disrupts endomembrane homeostasis and autophagy
Source: BMC Biol. 2020 Aug 28;18:107. doi: 10.1186/s12915-020-00837-w (PMC7453538; doi:10.1186/s12915-020-00837-w)
Supplement: Supplementary file 2 — Additional file 2: Table S1. Strains used in this study. Table S2. Plasmids constructed in this study. Table S3. DNA sequences of PCR primers. [file 12915_2020_837_MOESM2_ESM.pdf]

Table S1. Strains used in this study.

Name	Genotype
BY4741	*MATa his3Δ1 leu2Δ0 met15Δ0 ura3Δ0*
BY4742	*MATα his3Δ lleu2Δ0 ura3Δ0 lys2Δ*
TN124	*MATα leu2-3 112 trp1 ura3-52 pho8::pho8Δ60 pho13Δ::LEU2*
YLD9060	BY4742 *pho8Δ60 pho13Δ*
YLD9307	BY4742 *pho8Δ60 pho13Δ lro1Δ dga1Δ*
YLD9906	BY4741 *lro1Δ::kanMX4 dga1Δ::LEU2(K.l.) can1Δ::proCAN1-proMFA1-HIS5-terCAN1 URA3(K.l.) OM14-2EGFP::NAT*
YLD9907	BY4741 *can1Δ::proCAN1-proMFA1-HIS5-terCAN1 LEU2(K. l.) URA3(K.l.) OM14-2EGFP::NAT*
YLD9908	BY4741 *lro1Δ::kanMX4 dga1Δ::LEU2(K.l.) can1Δ::proCAN1-proMFA1-HIS5-terCAN1 URA3(K.l.) EMC1-2EGFP::NAT*
YLD9909	BY4741 *can1Δ::proCAN1-proMFA1-HIS5-terCAN1 LEU2(K.l.) URA3(K.l.) EMC1-2EGFP::NAT*
YLD9910	BY4741 *lro1Δ::kanMX4 dga1Δ::LEU2(K.l.) can1Δ::proCAN1-proMFA1-HIS5-terCAN1 URA3(K.l.) SEC7-2EGFP::NAT*
YLD9911	BY4741 *can1Δ::proCAN1-proMFA1-HIS5-terCAN1 LEU2(K.l.) URA3(K.l.) SEC7-2EGFP::NAT*
YLD9912	BY4741 *lro1Δ::kanMX4 dga1Δ::LEU2(K.l.) can1Δ::proCAN1-proMFA1-HIS5-terCAN1 URA3(K.l.) VRG4-2EGFP::NAT*
YLD9913	BY4741 *can1Δ::proCAN1-proMFA1-HIS5-terCAN1 LEU2(K.l.) URA3(K.l.) VRG4-2EGFP::NAT*
YLD9914	BY4741 *lro1Δ::kanMX4 dga1Δ::LEU2(K.l.) can1Δ::proCAN1-proMFA1-HIS5-terCAN1 URA3(K.l.) ATG8::NAT-ProATG8-GFP-ATG8*
YLD9915	BY4741 *can1Δ::proCAN1-proMFA1-HIS5-terCAN1 LEU2(K.l.) URA3(K.l.) ATG8::NAT-ProATG8-GFP-ATG8*
A571	BY4741 *ELO3::ELO3-mTagBFP2-HIS5(S.p.) proTPI1::pTPI1-GFP-HDEL-URA3(K.l.)*

A572	BY4741 *lro1Δ::kanMX4 ELO3::ELO3-mTagBFP2-HIS5(S.p.) proTPI1::pTPI1-GFP-HDEL-URA3(K. l.) dga1Δ::LEU2(K.l.)*
A165	TN124 *IRE1-2EGFP::kanMX4*
A169	TN124 *dga1Δ lro1Δ IRE1-2EGFP::kanMX4*
A229	TN124 *ELO3::ELO3-mTagBFP2-HIS5(S.p.) VRG4-2EGFP::NAT*
A230	TN124 *dga1Δ lro1Δ ELO3::ELO3-mTagBFP2-HIS5(S.p.) VRG4-2EGFP::NAT*
A356	BY4741 *LEU2(K.l.) HIS5(S.p.) ELO3::ELO3-mTagBFP2-HIS5(S.p.)*
A358	BY4741 *lro1Δ::kanMX4 dga1Δ::LEU2(K.l.) HIS5(S.p.) ELO3::ELO3-mTagBFP2-HIS5(S.p.)*
A227	TN124 *ELO3::ELO3-mTagBFP2-HIS5(S.p.) EMC1-2EGFP::NAT*
A228	TN124 *dga1Δ lro1Δ ELO3::ELO3-mTagBFP2-HIS5(S.p.) EMC1-2EGFP::NAT*
YLD9505	BY4742 *pho8Δ60 pho13Δ OM45-2EGFP::URA3(K.l.)*
YLD9506	BY4742 *pho8Δ60 pho13Δ lro1Δ dga1Δ OM45-2EGFP::URA3(K.l.)*
YLD9593	BY4742 *pho8Δ60 pho13Δ CIT1-2EGFP::URA3(K.l.)*
YLD9594	BY4742 *pho8Δ60 pho13Δ lro1Δ dga1Δ CIT1-2EGFP::URA3(K.l.)*
YLD9595	BY4742 *pho8Δ60 pho13Δ ABF2-2EGFP::URA3(K.l.)*
YLD9596	BY4742 *pho13Δ lro1Δ dga1Δ ABF2-2EGFP::URA3(K.l.)*
YLD9526	BY4742 *pho8Δ60 pho13Δ COX4::COX4-2DsRed-TRP1*
YLD9527	BY4742 *pho8Δ60 pho13Δ lro1Δ dga1Δ COX4::COX4-2DsRed-TRP1*
YLD9561	BY4742 *pho8Δ60 pho13Δ ACH1-2EGFP::URA3(K.l.)*

YLD9562	BY4742 *pho8Δ60 pho13Δ lro1Δ dga1Δ ACH1-2EGFP::URA3(K.l.)*
YLD9597	BY4742 *pho8Δ60 pho13Δ AFG3-2EGFP::URA3(K.l.)*
YLD9598	BY4742 *pho8Δ60 pho13Δ lro1Δ dga1Δ AFG3-2EGFP::URA3(K.l.)*
YLD9709	BY4742 *pho8Δ60 pho13Δ SYS1-2EGFP::URA3(K.l.)*
YLD9710	BY4742 *pho8Δ60 pho13Δ lro1Δ dga1Δ SYS1-2EGFP::URA3(K.l.)*
YLD9481	BY4742 *pho8Δ60 pho13Δ CHS5::1K-CHS5-EGFP-URA3(K.l.)*
YLD9482	BY4742 *pho8Δ60 pho13Δ lro1Δ dga1Δ CHS5::CHS5-EGFP-URA3(K.l.)*
YLD9487	BY4742 *pho8Δ60 pho13Δ SNF7::SNF7-EGFP-URA3(K.l.)*
YLD9488	BY4742 *pho8Δ60 pho13Δ lro1Δ dga1Δ SNF7::SNF7-EGFP-URA3(K.l.)*
YLD9466	BY4742 *pho8Δ60 pho13Δ NAB2::NAB2-EGFP-URA3(K.l.)*
YLD9467	BY4742 *pho8Δ60 pho13Δ lro1Δ dga1Δ NAB2::NAB2-EGFP-URA3(K.l.)*
A186	TN124 *ELO3::ELO3-mTagBFP2-TRP1(K.l.) trp1Δ::NAT-proLYS2-GFP-PKCδ(C1a+C1b) VPH1::VPH1-mCherry-URA3(K.l.)*
A188	TN124 *dga1Δ lro1Δ ELO3::1K-ELO3-mTagBFP2-TRP1(K.l.) trp1Δ::NAT-proLYS2-GFP-PKCδ(C1a+C1b) VPH1::VPH1-mCherry-URA3(K.l.)*
A291	TN124 *PEX1::PEX1-2GFP-URA3(K.l.)*
A292	TN124 *dga1Δ lro1Δ PEX1::PEX1-2GFP-URA3(K.l.)*
YLD9478	BY4742 *pho8Δ60 pho13Δ TGL3::TGL3-EGFP-URA3(K.l.)*
YLD9479	BY4742 *pho8Δ60 pho13Δ lro1Δ dga1Δ TGL3::TGL3-EGFP-URA3(K.l.)*
YLD9931	TN124 *TRP1(K.l.) URA3(K.l.) OM14-2EGFP::NAT*

YLD9932	TN124 *dga1Δ::URA3(K.l.) lro1Δ::TRP1(K.l.) OM14-2EGFP::NAT*
YLD9937	TN124 *slc1Δ::URA3(K.l.) TRP1(K.l.) OM14-2EGFP::NAT*
YLD9938	TN124 *slc1Δ::URA3(K.l.) lro1Δ::TRP1(K.l.) dga1Δ::hyg OM14-2EGFP::NAT*
YLD9941	TN124 *TRP1(K.l.) URA3(K.l.) EMC1-2EGFP::NAT*
YLD9942	TN124 *dga1Δ::URA3(K.l.) lro1Δ::TRP1(K.l.) EMC1-2EGFP::NAT*
YLD9947	TN124 *slc1Δ::URA3(K.l.) TRP1(K.l.) EMC1-2EGFP::NAT*
YLD9948	TN124 *slc1Δ::URA3(K.l.) lro1Δ::TRP1(K.l.) dga1Δ::hyg EMC1-2EGFP::NAT*
YLD9961	TN124 *TRP1(K. l.) URA3(K.l.) SEC7-2EGFP::NAT*
YLD9962	TN124 *dga1Δ::URA3(K.l.) lro1Δ::TRP1(K.l.) SEC7-2EGFP::NAT*
YLD9967	TN124 *slc1Δ::URA3(K.l.) TRP1(K.l.) SEC7-2EGFP::NAT*
YLD9968	TN124 *slc1Δ::URA3(K.l.) lro1Δ::TRP1(K.l.) dga1Δ::hyg SEC7-2EGFP::NAT*
YLD9971	TN124 *TRP1(K.l. URA3(K.l.) VRG4-2EGFP::NAT*
YLD9972	TN124 *dga1Δ::URA3(K.l.) lro1Δ::TRP1(K.l.) VRG4-2EGFP::NAT*
YLD9977	TN124 *slc1Δ::URA3(K.l.) TRP1(K.l.) VRG4-2EGFP::NAT*
YLD9978	TN124 *slc1Δ::URA3(K.l.) lro1Δ::TRP1(K.l.) dga1Δ::hyg VRG4-2EGFP::NAT*
YLD9981	TN124 *TRP1(K.l.) URA3(K.l.) ATG8::NAT-ProATG8-GFP-ATG8*
YLD9982	TN124 *dga1Δ::URA3(K.l.) lro1Δ::TRP1(K.l.) ATG8::NAT-ProATG8-GFP-ATG8*
YLD9987	TN124 *slc1Δ::URA3(K.l.) TRP1(K.l.) ATG8::NAT-ProATG8-GFP-ATG8*

YLD9988	TN124 *slc1Δ::URA3(K.l.) lro1Δ::TRP1(K.l.) dga1Δ::hyg ATG8::NAT-ProATG8-GFP-ATG8*
A966	BY4741 *lro1Δ::kanMX4 ELO3::ELO3-mTagBFP2-HIS5(S.p.) faa1Δ::URA3(K.l.) faa4Δ::LEU2(K.l.) dga1Δ::MET15 OM14-2EGFP::NAT*
A967	BY4741 *lro1Δ::kanMX4 ELO3::ELO3-mTagBFP2-HIS5(S.p.) faa1Δ::URA3(K.l.) faa4Δ::LEU2(K.l.) dga1Δ::MET15 EMC1-2EGFP::UG74*
A968	BY4741 *lro1Δ::kanMX4 ELO3::ELO3-mTagBFP2-HIS5(S.p.) faa1Δ::URA3(K.l.) faa4Δ::LEU2(K.l.) dga1Δ::MET15 SEC7-2EGFP::UG74*
A969	BY4741 *lro1Δ::kanMX4 ELO3::ELO3-mTagBFP2-HIS5(S.p.) faa1Δ::URA3(K.l.) faa4Δ::LEU2(K.l.) dga1Δ::MET15 VRG4-2EGFP::UG74*
A970	BY4741 *lro1Δ::kanMX4 ELO3::ELO3-mTagBFP2-HIS5(S.p.) faa1Δ::URA3(K.l.) faa4Δ::LEU2(K.l.) dga1Δ::MET15 ATG8::NAT-ProATG8-GFP-ATG8*
A971	BY4741 *lro1Δ::kanMX4 ELO3::ELO3-mTagBFP2-HIS5(S.p.) faa1Δ::URA3(K.l.) faa4Δ::LEU2(K.l.) dga1Δ::MET15 OM14-2EGFP::NAT LRO1::LRO1-DGA1-hyg*
A972	BY4741 *lro1Δ::kanMX4 ELO3::ELO3-mTagBFP2-HIS5(S.p.) faa1Δ::URA3(K.l.) faa4Δ::LEU2(K.l.) dga1Δ::MET15 EMC1-2EGFP::UG74 LRO1::LRO1-DGA1-hyg*
A973	BY4741 *lro1Δ::kanMX4 ELO3::ELO3-mTagBFP2-HIS5(S.p.) faa1Δ::URA3(K.l.) faa4Δ::LEU2(K.l.) dga1Δ::MET15 SEC7-2EGFP::UG74 LRO1::LRO1-DGA1-hyg*
A974	BY4741 *lro1Δ::kanMX4 ELO3::ELO3-mTagBFP2-HIS5(S.p.) faa1Δ::URA3(K.l.) faa4Δ::LEU2(K.l.) dga1Δ::MET15 VRG4-2EGFP::UG74 LRO1::LRO1-DGA1-hyg*
A975	BY4741 *lro1Δ::kanMX4 ELO3::ELO3-mTagBFP2-HIS5(S.p.) faa1Δ::URA3(K.l.) faa4Δ::LEU2(K.l.) dga1Δ::MET15 ATG8::NAT-ProATG8-GFP-ATG8 LRO1::LRO1-DGA1-hyg*

A963	BY4741 *lro1Δ::kanMX4 ELO3::ELO3-mTagBFP2-HIS5(S.p.) OM14-2EGFP::NAT faa4Δ::LEU2(K.l.) dga1Δ::URA3(K.l.)*
A964	BY4741 *lro1Δ::kanMX4 ELO3::ELO3-mTagBFP2-HIS5(S.p.) faa1Δ::URA3(K.l.) dga1Δ::LEU2(K.l.) OM14-2EGFP::NAT*
YLD4008	TN124 *TRP1 URA3(K.l.)*
YLD486	TN124 *lro1Δ::URA3(K.l.) dga1::TRP1(K.l.)*
A198	TN124 *yeh1Δ::URA3(K.l.) TRP1*
YLD6071	TN124 *yeh2Δ::URA3(K.l.) TRP1*
YLD6072	TN124 *ldh1Δ::URA3(K.l.) TRP1*
YLD6073	TN124 *ayr1Δ::URA3(K.l.) TRP1*
YLD6074	TN124 *ldb16Δ::URA3(K.l.) TRP1*
YLD6075	TN124 *ice2Δ::URA3(K.l.) TRP1*
YLD6076	TN124 *ldh1Δ::URA3(K.l.) ayr1Δ::TRP1(K.l.)*
YLD6078	TN124 *ldb16Δ::URA3(K.l.) ice2Δ::TRP1(K.l.)*
YLD9981	TN124 *TRP1 URA3(K.l.) ATG8::NAT-ProATG8-GFP-ATG8*
A201	TN124 *yeh1Δ::TRP1(K.l.) URA3(K.l.) ATG8::NAT-ProATG8-GFP-ATG8*
A202	TN124 l*db16Δ::URA3(K.l.) TRP1 ATG8::NAT-ProATG8-GFP-ATG8*
A203	TN124 *ice2Δ::URA3(K.l.) TRP1 ATG8::NAT-ProATG8-GFP-ATG8*
A204	TN124 *ldh1Δ::URA3(K.l.) ayr1Δ::TRP1(K.l.) ATG8::NAT-ProATG8-GFP-ATG8*
A206	TN124 *ldb16Δ::UG72 ice2Δ::TRP1(K.l.) ATG8::NAT-ProATG8-GFP-ATG8*

YLD9934	TN124 *dgk1Δ::TRP1(K.l.) URA3(K.l.) OM14-2EGFP::NAT*
YLD9935	TN124 *dgk1Δ::TRP1(K.l.) lro1Δ::URA3(K.l.) dga1Δ::hyg OM14-2EGFP::NAT*
YLD9944	TN124 *dgk1Δ::TRP1(K.l.) URA3(K.l.) EMC1-2EGFP::NAT*
YLD9945	TN124 *dgk1Δ::TRP1(K.l.) lro1Δ::URA3(K.l.) dga1Δ::hyg EMC1-2EGFP::NAT*
YLD9964	TN124 *dgk1Δ::TRP1(K.l.) URA3(K.l.) SEC7-2EGFP::NAT*
YLD9965	TN124 *dgk1Δ::TRP1(K.l.) lro1Δ::URA3(K.l.) dga1Δ::hyg SEC7-2EGFP::NAT*
YLD9974	TN124 *dgk1Δ::TRP1(K.l.) URA3(K.l.) VRG4-2EGFP::NAT*
YLD9975	TN124 *dgk1Δ::TRP1(K.l.) lro1Δ::URA3(K.l.) dga1Δ::hyg VRG4-2EGFP::NAT*
YLD9984	TN124 *dgk1Δ::TRP1(K.l.) URA3(K.l.) ATG8::NAT-ProATG8-GFP-ATG8*
YLD9985	TN124 *dgk1Δ::TRP1(K.l.) lro1Δ::URA3(K.l.) dga1Δ::hyg ATG8::NAT-ProATG8-GFP-ATG8*
YLD6332	TN124 *OM14-2EGFP::NAT*
YLD6333	TN124 *EMC1-2EGFP::NAT*
YLD6334	TN124 *SEC7-2EGFP::NAT*
YLD6340	TN124 *OM14-2EGFP::NAT pah1Δ::TRP1(K.l.)*
YLD6341	TN124 *EMC1-2EGFP::NAT pah1Δ::TRP1(K.l.)*
YLD6342	TN124 *SEC7-2EGFP::NAT pah1Δ::TRP1(K.l.)*
A163	TN124 *VRG4-2EGFP::NAT*
A171	TN124 *VRG4-2EGFP::NAT pah1Δ::TRP1(K.l.)*

YLD6331	TN124 *ATG8::NAT-ProATG8-GFP-ATG8*
YLD6339	TN124 *ATG8::NAT-ProATG8-GFP-ATG8 pah1Δ::TRP1(K.l.)*
A162	TN124 *SNF7::1K-SNF7-EGFP-URA3(K.l.)*
A170	TN124 *SNF7::1K-SNF7-EGFP-URA3(K.l.) pah1Δ::TRP1(K.l.)*
A178	TN124 *NAB2::1K-NAB2-mTagBFP2-TRP1(K.l.)*
A209	TN124 *NAB2::1K-NAB2-mTagBFP2-TRP1(K.l.) pah1Δ::URA3(K.l.)*
A158	TN124 *dga1Δ lro1Δ trp1Δ::NAT-proLYS2-GFP-PKCδ(C1a+C1b) VPH1::VPH1-mCherry-TRP1(K.l.) LRO1::LRO1-DGA1-URA3(K.l.)*
A208	TN124 *dga1Δ lro1Δ trp1Δ::NAT-proLYS2-GFP-PKCδ(C1a+C1b) VPH1::1K-VPH1-mCherry-TRP1(K.l.) proLRO1::LRO1-DGA1-URA3(K.l.) pah1Δ::hyg*
A401	BY4741 *lro1Δ::kanMX DGA1::hyg-GAL-DGA1 OM14-2EGFP::NAT pah1Δ::URA3(K.l.)*
A402	BY4741 *lro1Δ::kanMX DGA1::hyg-GAL-DGA1 EMC1-2EGFP::NAT pah1Δ::URA3(K.l.)*
A403	BY4741 *lro1Δ::kanMX DGA1::hyg-GAL-DGA1 SEC7-2EGFP::NAT pah1Δ::URA3(K.l.)*
A404	BY4741 *lro1Δ::kanMX DGA1::hyg-GAL-DGA1 VRG4-2EGFP::NAT pah1Δ::URA3(K.l.)*
YLD9951	TN124 *TRP1(K.l.) URA3(K.l.) trp1Δ::NAT-proLYS2-GFP-PKCδ(C1a+C1b)*
YLD9952	TN124 *dga1Δ::URA3(K.l.) lro1Δ::TRP1(K.l.) trp1Δ::NAT-proLYS2-GFP-PKCδ(C1a+C1b)*
A210	TN124 *PKC1-2GFP::TRP1(K.l.)*
A211	TN124 *dga1Δ lro1Δ PKC1-2GFP::TRP1(K.l.)*
A293	TN124 *VPH1::1K-VPH1-mTagBFP2-TRP1(K.l.) trp1Δ::NAT-proLYS2-GFP-PKCδ(C1a+C1b)*

A294	TN124 *dga1Δ lro1Δ VPH1::1K-VPH1-mTagBFP2-TRP1(K.l.) trp1Δ::NAT-proLYS2-GFP-PKCδ(C1a+C1b)*
A186	TN124 *ELO3::1K-ELO3-mTagBFP2-TRP1(K.l.) trp1Δ::NAT-proLYS2-GFP-PKCδ(C1a+C1b) VPH1::VPH1-mCherry-URA3(K.l.)*
A188	TN124 *dga1Δ lro1Δ ELO3::1K-ELO3-mTagBFP2-TRP1(K.l.) trp1Δ::NAT-proLYS2-GFP-PKCδ(C1a+C1b) VPH1::VPH1-mCherry-URA3(K.l.)*
A297	TN124 *SEC26::SEC26-mTagBFP2-TRP1(K.l.) trp1Δ::NAT-proLYS2-GFP-PKCδ(C1a+C1b)*
A298	TN124 *dga1Δ lro1Δ SEC26::SEC26-mTagBFP2-TRP1(K.l.) trp1Δ::NAT-proLYS2-GFP-PKCδ(C1a+C1b)*
A301	TN124 *SEC7::SEC7-mTagBFP2-trp trp1Δ::NAT-proLYS2-GFP-PKCδ(C1a+C1b)*
A302	TN124 *dga1Δ lro1Δ SEC7::SEC7-mTagBFP2-trp trp1Δ::NAT-proLYS2-GFP-PKCδ(C1a+C1b)*
A299	TN124 *VPS4::VPS4-mTagBFP2-TRP1(K.l.) trp1Δ::NAT-proLYS2-GFP-PKCδ(C1a+C1b)*
A300	TN124 *dga1Δ lro1Δ VPS4::VPS4-mTagBFP2-TRP1(K.l.) trp1Δ::NAT-proLYS2-GFP-PKCδ(C1a+C1b)*
A187	TN124 *NAB2::NAB2-mTagBFP2-TRP1(K.l.) trp1Δ::NAT-proLYS2-GFP-PKCδ(C1a+C1b) SNF7::SNF7-mCherry-URA3(K.l.)*
A189	TN124 *dga1Δ lro1Δ NAB2::NAB2-mTagBFP2-TRP1(K.l.) trp1Δ::NAT-proLYS2-GFP-PKCδ(C1a+C1b) SNF7::SNF7-mCherry-URA3(K.l.)*
A339	TN124 *proTPI1::pTPI1-mTagBFP2-SKL-TRP1(K.l.) trp1Δ::NAT-proLYS2-GFP-PKCδ(C1a+C1b)*
A340	TN124 *dga1Δ lro1Δ proTPI1::pTPI1-mTagBFP2-SKL-TRP1(K.l.) trp1Δ::NAT-proLYS2-GFP-PKCδ(C1a+C1b)*
A465	BY4741 *lro1Δ::kanMX4 ELO3::ELO3-mTagBFP2-HIS5(S.p.) OM14-2EGFP::NAT bck1Δ::LEU2(K.l.) dga1Δ::URA3(K.l.)*
A466	BY4741 *lro1Δ::kanMX4 ELO3::ELO3-mTagBFP2-HIS5(S.p.) OM14-2EGFP::NAT slt2Δ::LEU2(K.l.) dga1Δ::URA3(K.l.)*
A467	BY4741 *lro1Δ::kanMX4 ELO3::ELO3-mTagBFP2-HIS5(S.p.) OM14-2EGFP::NAT mkk1Δ::LEU2(K.l.) dga1Δ::URA3(K.l.)*

A468	BY4741 *lro1Δ::kanMX4 ELO3::ELO3-mTagBFP2-HIS5(S.p.) OM14-2EGFP::NAT mkk2Δ::LEU2(K.l.) dga1Δ::URA3(K.l.)*
A91	TN124 *opi1Δ::TRP1(K.l.) URA3(K.l.) OM14-2EGFP::NAT*
YLD6910	TN124 *dga1Δ::URA3(K.l.) opi1Δ::TRP1(K.l.) lro1Δ::hyg OM14-2EGFP::NAT*
A92	TN124 *opi1Δ::TRP1(K.l.) URA3(K.l.) EMC1-2EGFP::NAT*
YLD6810	TN124 *dga1Δ::URA3(K.l.) opi1Δ::TRP1(K.l.) lro1Δ::hyg EMC1-2EGFP::NAT*
A94	TN124 *opi1Δ::TRP1(K.l.) URA3(K.l.) SEC7-2EGFP::NAT*
YLD6610	TN124 *dga1Δ::URA3(K.l.) opi1Δ::TRP1(K.l.) lro1Δ::hyg SEC7-2EGFP::NAT*
A95	TN124 *opi1Δ::TRP1(K.l.) URA3(K.l.) VRG4-2EGFP::NAT*
A97	TN124 *dga1Δ::URA3(K.l.) opi1Δ::TRP1(K.l.) lro1Δ::hyg VRG4-2EGFP::NAT*
A96	TN124 *opi1Δ::TRP1(K.l.) URA3(K.l.) ATG8::NAT-ProATG8-GFP-ATG8*
A98	TN124 *dga1Δ::URA3(K.l.) opi1Δ::TRP1(K.l.) lro1Δ::hyg ATG8::NAT-ProATG8-GFP-ATG8*
YLD4008	TN124 *TRP1(K.l.) URA3(K.l.)*
YLD486	TN124 *lro1Δ::URA3(K.l.) dga1Δ::TRP1(K.l.)*
YLD7018	TN124 *ino2Δ::TRP1(K.l.) URA3(K.l.)*
YLD7019	TN124 *ino4Δ::TRP1(K.l.) URA3(K.l.)*
YLD7020	TN124 *opi1Δ::TRP1(K.l.) URA3(K.l.)*
YLD6028	TN124 *TRP1(K.l.) pah1Δ::URA3(K.l.)*
YLD6016	TN124 *dgk1Δ::TRP1(K.l.) pah1Δ::URA3(K.l.)*

YLD6062	TN124 *ino2Δ::TRP1(K.l.) pah1Δ::URA3(K.l.)*
YLD6063	TN124 *ino4Δ::TRP1(K.l.) pah1Δ::URA3(K.l.)*
YLD6064	TN124 *opi1Δ::TRP1(K.l.) pah1Δ::URA3(K.l.)*
YLD6065	TN124 *slc1Δ::TRP1(K.l.) pah1Δ::URA3(K.l.)*
YLD7028	TN124 opi*1Δ::TRP1(K.l.) dga1Δ::URA3(K.l.) lro1Δ::hyg*
YLD9951	TN124 *TRP1(K.l.) URA3(K.l.) trp1Δ::NAT-proLYS2-GFP-PKCδ(C1a+C1b)*
YLD9952	TN124 *dga1Δ::URA3(K.l.) lro1Δ::TRP1(K.l.) trp1Δ::NAT-proLYS2-GFP-PKCδ(C1a+C1b)*
YLD9954	TN124 *dgk1Δ::TRP1(K.l.) URA3(K.l.) trp1Δ::NAT-proLYS2-GFP-PKCδ(C1a+C1b)*
YLD9955	TN124 *dgk1Δ::TRP1(K.l.) lro1Δ::URA3(K.l.) dga1Δ::hyg trp1Δ::NAT-proLYS2-GFP-PKCδ(C1a+C1b)*
YLD9957	TN124 *slc1Δ::URA3(K.l.) TRP1(K.l.) trp1Δ::NAT-proLYS2-GFP-PKCδ(C1a+C1b)*
YLD9958	TN124 *slc1Δ::URA3(K.l.) lro1Δ::TRP1(K.l.) dga1Δ::hyg trp1Δ::NAT-proLYS2-GFP-PKCδ(C1a+C1b)*
A93	TN124 *opi1Δ::TRP1(K.l.) URA3(K.l.) trp1Δ::NAT-proLYS2-GFP-PKCδ(C1a+C1b)*
YLD6710	TN124 *dga1Δ::URA3(K.l.) opi1Δ::TRP1(K.l.) lro1Δ::hyg trp1Δ::NAT-proLYS2-GFP-PKCδ(C1a+C1b)*
YLD6335	TN124 *trp1Δ::NAT-proLYS2-GFP-PKCδ(C1a+C1b)*
YLD6343	TN124 *trp1Δ::NAT-proLYS2-GFP-PKCδ(C1a+C1b) pah1Δ::TRP1(K.l.)*
A1131	BY4741 *lro1Δ::kana dga1Δ::LEU2 proCAN1-AvrII-proMFA1-HIS5-terCAN1::HIS5 URA3 Emc1-2EGFP::NAT ADH1::UG75-proADH1-DGK1-3HA*
A1132	BY4741 *lro1Δ::kana dga1Δ::LEU2 proCAN1-AvrII-proMFA1-HIS5-terCAN1::HIS5 URA3 Emc1-2EGFP::NAT ADH1::UG75-proADH1-CDS1-3HA*

A1133	BY4741 *lro1Δ::kana dga1Δ::LEU2 proCAN1-AvrII-proMFA1-HIS5-terCAN1::HIS5 URA3 Emc1-2EGFP::NAT ADH1::UG75-proADH1-PAH1-3HA*
A1134	BY4741 *proCAN1-AvrII-proMFA1-HIS5-terCAN1::HIS5 LEU2 URA3 Emc1-2EGFP::NAT ADH1::UG75-proADH1-DGK1-3HA*
A1135	BY4741 *proCAN1-AvrII-proMFA1-HIS5-terCAN1::HIS5 LEU2 URA3 Emc1-2EGFP::NAT ADH1::UG75-proADH1-CDS1-3HA*
A1136	BY4741 *proCAN1-AvrII-proMFA1-HIS5-terCAN1::HIS5 LEU2 URA3 Emc1-2EGFP::NAT ADH1::*UG75-proADH1-PAH1-3HA
A1137	BY4741 *lro1Δ::kana dga1Δ::LEU2 proCAN1-AvrII-proMFA1-HIS5-terCAN1::HIS5 URA3 ATG8::NAT-ProATG8-GFP-ATG8 ADH1::UG75-proADH1-DGK1-3HA*
A1138	BY4741 *lro1Δ::kana dga1Δ::LEU2 proCAN1-AvrII-proMFA1-HIS5-terCAN1::HIS5 URA3 ATG8::NAT-ProATG8-GFP-ATG8 ADH1::UG75-proADH1-CDS1-3HA*
A1139	BY4741 *lro1Δ::kana dga1Δ::LEU2 proCAN1-AvrII-proMFA1-HIS5-terCAN1::HIS5 URA3 ATG8::NAT-ProATG8-GFP-ATG8 ADH1::UG75-proADH1-PAH1-3HA*
A1140	BY4741 *proCAN1-AvrII-proMFA1-HIS5-terCAN1 LEU2 URA3 ATG8::NAT-ProATG8-GFP-ATG8 ADH1::UG75-proADH1-DGK1-3HA*
A1141	BY4741 *proCAN1-AvrII-proMFA1-HIS5-terCAN1 LEU2 URA3 ATG8::NAT-ProATG8-GFP-ATG8 ADH1::UG75-proADH1-CDS1-3HA*
A1142	BY4741 *proCAN1-AvrII-proMFA1-HIS5-terCAN1 LEU2 URA3 ATG8::NAT-ProATG8-GFP-ATG8 ADH1::UG75-proADH1-PAH1-3HA*
A1144	TN124 *dga1Δ lro1Δ trp1Δ::NAT-proLYS2-GFP-PKCδ(C1a+C1b) CU-2Dsred-Atg8::TRP1*
A1146	TN124 *dga1Δ lro1Δ trp1Δ::NAT-proLYS2-GFP-PKCδ(C1a+C1b) CU-2Dsred-Atg8::TRP1 LRO1-DGA1::KANA*
A1151	TN124 *LYS2::proLYS2-GFP-PKCbeta-TRP1*
A1152	TN124 *dga1Δ lro1Δ LYS2::proLYS2-GFP-PKCbeta-TRP1*
A1155	BY4741 atg1::ATG5-2EGFP-URA3

A1156	BY4741 *lro1Δ::kana dga1Δ::LEU2* atg1::ATG5-2EGFP-URA3
A1157	BY4741 atg5::ATG5-2EGFP-URA3
A1158	BY4741 *lro1Δ::kana dga1Δ::LEU2* atg5::ATG5-2EGFP-URA3
A1159	BY4741 atg14::ATG5-2EGFP-URA3
A1160	BY4741 *lro1Δ::kana dga1Δ::LEU2* atg14::ATG5-2EGFP-URA3
A1161	BY4741 *sec16ts emc1::EMC1-GFP-URA3*
A1162	BY4741 *sec16ts vrg4::vrg4-GFP-URA3*
A1163	BY4741 *sec16ts sec7::sec7-2EGFP-NAT*
A1164	BY4741 *sec16ts ATG8::NAT-ProATG8-GFP-ATG8*
A1165	BY4741 *sec16ts atg1::ATG1-2EGFP-URA3*
A1166	BY4741 *emc1::EMC1-2EGFP-NAT ire1Δ::URA3*
A1167	BY4741 *lro1Δ::kana DGA1::Hygromycin-GAL-DGA1 emc1::EMC1-2EGFP-NAT ire1Δ::URA3*
A1168	BY4741 *emc1::EMC1-2EGFP-NAT ice2Δ::URA3*
A1169	BY4741 *lro1Δ::kana DGA1::Hygromycin-GAL-DGA1 emc1::EMC1-2EGFP-NAT ice2Δ::URA3*
A1170	BY4741 *emc1::EMC1-2EGFP-NAT fld1Δ:: URA3*
A1171	BY4741 *lro1Δ::kana DGA1::Hygromycin-GAL-DGA1 emc1::EMC1-2EGFP-NAT fld1Δ:: URA3*
YCH717	TN124 *TPI1::*pTPI1-EMC1-GFP-APEX2-URA3
A1148	TN124 *dga1Δ lro1Δ TPI1::*pTPI1-EMC1-GFP-APEX2-URA3

Table S2. Plasmids constructed in this study.

Plasmid	Parental plasmid	Restriction sites for plasmid construction	Primers for insert amplification	Linearization sites for genome integration	Genome integration locus
OM14-2EGFP-ter-NAT	UG74-2EGFP	HindIII SacII	OM14F/OM14R	NheI AflII	C terminal
UG75-pADH1-DGK1-3HA	UG75	SacII	ADH1F/ADH1R	SphI	ADH1 promoter
UG75-pADH1-CDS1-3HA	UG75	SacII	ADH1F/ADH1R	SphI	ADH1 promoter
UG75-pADH1-PAH1-3HA	UG75	SacII	ADH1F/ADH1R	SphI	ADH1 promoter
UG76-pLYS2-GFP-PKCbeta	UG76	HindIII	PKCbetaF/PKCbetaR	EcoRI	LYS2 promoter
EMC1-2EGFP-ter-NAT	UG74-2EGFP	HindIII SacII	EMC1F/EMC1R	BspEI AfeI	C terminal
SEC7-2EGFP-ter-NAT	UG74-2EGFP	HindIII SacII	SEC7F/SEC7R	SnaBI AflII	C terminal
VRG4-2EGFP-ter-NAT	UG74-2EGFP	HindIII SacII	VRG4F/VRG4R	AseI EcoRI	C terminal
IRE1-2EGFP-ter-kanMX4	UG6-2EGFP	HindIII SacII	IRE1F/IRE1R	PspOMI HindIII	C terminal
CIT1-2EGFP-ter-URA3	UG72-2EGFP	HindIII SacII	CIT1F/CIT1R	Cit1 PmlI	C terminal
ABF2-2EGFP-ter-URA3	UG72-2EGFP	HindIII SacII	ABF2F/ABF2R	SnaBI AflII	C terminal
ACH1-2EGFP-ter-URA3	UG74-2EGFP	HindIII SacII	ACH1F/ACH1R	PmlI SacII	C terminal
AFG3-2EGFP-ter-URA3	UG72-2EGFP	HindIII SacII	AFG3F/AFG3R	AgeI PvuI	C terminal
SYS1-2EGFP-ter-URA3	UG72-2EGFP	HindIII SacII	SYS1F/SYS1R	NurI SnaBI	C terminal

SEC7-2katushka-ter-Hygromycin	UG75-2EGFP	HindIII SacII	SEC7F/SEC7R	SnaBI AflII	C terminal
PKC1-2GFP-ter-TRP1	UG76-2GFP	HindIII SacII	PKC1F/PKC1R	ClaI AgeI	C terminal
trp1D-proLYS2-GFP-PKCδ(C1a+C1b)-NAT	TRP-UG74	PspOMI SacII	Lys2F/lys2R/GFPF/GFPR	SacI BstBI	*TRP1 promoter and terminator*[1]
GAL-Aur1-2GFP-Hygromycin	GAL-UG75	SacII	Aur1GF/Aur1GR	PspOMI	*AUR1* ORF
pTPI1-EMC1-GFP-APEX2-URA3	UG72-EMC1-GFP	BmtI AvrII	APEX2-Clhn-F/Clhn-APEX2-R	SphI	*TPI1 promoter*

1. Its integration replaces *TRP1* ORF with the GFP-PKCδ-NAT construct, resulting in *trp1Δ*.

Table S3. DNA sequences of PCR primers.

Primer name	Sequence
OM14F1	CGCGGCCGCCAGCTGAAGCTTGCTAAACACGACAGTAACGC
OM14R1	GACCTGCAGCGTACGAAGCTTTCTTGTCGTATCTGGAGTAG
OM14F2	TAGTGGCCTATGCGGCCGCGGCATCCTTTCACACACTCACAC
OM14R2	GGGAGACCGGCAGATCCGCGGCCTGCTTAGTTCTATGATGTTC
EMC1F1	CGCGGCCGCCAGCTGAAGCTTCATTTACTGTTCAAATTGAATC
EMC1R1	GACCTGCAGCGTACGAAGCTTTAATTAGCCATTGGGATTTC
EMC1F2	TAGTGGCCTATGCGGCCGCGGTAGATATTTACATACACATTAC
EMC1R2	GGGAGACCGGCAGATCCGCGGAGGTAGCGCTTTTATTAAGAG
SEC7F1	CGCGGCCGCCAGCTGAAGCTTGATATTGCAAAACGTATCTA
SEC7R1	GACCTGCAGCGTACGAAGCATCAGTAGAAAGGTATAATTC
SEC7F2	TAGTGGCCTATGCGGCCGCGGCAGATTAAAATATGCTTAGTTG
SEC7R2	GGGAGACCGGCAGATCCGCGGCCAAAAGTTGCCTGTCCAAAG
VRG4F1	CGCGGCCGCCAGCTGAAGCTTCAAGCCTATTTCTTGTTGCC
VRG4R1	GACCTGCAGCGTACGAAGCTTTACGTAAAGGTTGGGCTTG
VRG4F2	TAGTGGCCTATGCGGCCGCGGTGAGAACTTACGGGGGGTGC
VRG4R2	GGGAGACCGGCAGATCCGCGGCTTGGACCGAATCATTACTAG

IRE1F1	CGCGGCCGCCAGCTGAAGCTTATTACAGAAAGAGTATAATC
IRE1R1	GACCTGCAGCGTACGAAGCTGAATACAAAAATTCACGTA
IRE1F2	TAGTGGCCTATGCGGCCGCGGTGTTCATGCCCCTCTGCTTC
IRE1R2	GGGAGACCGGCAGATCCGCGGCTGGTATCAACTGGAGCTTG
CIT1F1	CGCGGCCGCCAGCTGAAGCTTCGGTAAAACCGTTATTGGTG
CIT1R1	GACCTGCAGCGTACGAAGCGTTCTTACTTTCGATTTTCT
CIT1F2	TAGTGGCCTATGCGGCCGCGGCAGGGTATGCGACTATTCAAAC
CIT1R2	GGGAGACCGGCAGATCCGCGGCTATGTAAGAAAAAAGTAATG
ABF2F1	CGCGGCCGCCAGCTGAAGCTTAACGTGGAACAGATTCGCTATC
ABF2R1	GACCTGCAGCGTACGAAGCGTTGAGAGGGTAGCGAGCAT
ABF2F2	TAGTGGCCTATGCGGCCGCGGAGACCGCGGTACTCTCACAATG
ABF2R2	GGGAGACCGGCAGATCCGCGGAGATTTCAGCGAAGATCTGA
ACH1F1	CGCGGCCGCCAGCTGAAGCTTATGACAATTTCTAATTTGTTA
ACH1R1	GACCTGCAGCGTACGAAGCGTCAACTGGTTCCCAGCTGTC
ACH1F2	TAGTGGCCTATGCGGCCGCGGGTTTGTGCGCAAACCGAGAG
ACH1R2	GGGAGACCGGCAGATCCGCGGGTCGTTATTTTCATGCCATTC
AFG3F1	CGCGGCCGCCAGCTGAAGCTTGAAGAACCCAGGTAAGTACAC
AFG3R1	CCTGCAGCGTACGAAGCATTTGTTGCTGCAGGTGCCTC

AFG3F2	TAGTGGCCTATGCGGCCGCGGGAAATAAAACGGTGGCTTCTTC
AFG3R2	GGGAGACCGGCAGATCCGCGGATGGAATTGAGAAAACAGTG
SYS1F1	CGCGGCCGCCAGCTGAAGCTTCCAGATATAAGGAAGTGTAT
SYS1R1	GACCTGCAGCGTACGAAGCTATTTGGCTTTCTAAGTCTT
SYS1F2	TAGTGGCCTATGCGGCCGCGGTGAGAGGAGATGAAACAACA
SYS1R2	GGGAGACCGGCAGATCCGCGGTCAGATTGCAATGAGTAGTGA
PKC1F1	CGCGGCCGCCAGCTGAAGCTTGCTATTATCAATAGGAAAGAAG
PKC1R1	GACCTGCAGCGTACGAAGCTAAATCCAAATCATCTGGCA
PKC1F2	TAGTGGCCTATGCGGCCGCGGTGAAGAAAAGGTCATGCCATG
PKC1R2	GGGAGACCGGCAGATCCGCGGGACTTTGTAGTCAATGAAAG
Lys2F	GCTATACTGGGTACCGGGCCCCATCTGTTGTGGCTTTATCAC
lys2R	CCTTTAGACATGTTACTAGTTAGTGAGTAACTCTGTGATATC
GFPF	ACTAGTAACATGTCTAAAGGTG
GFPR	CTCAGTAATAACCCACCGCGGCTAACACAGGTTGGCCACCTTC
Aur1GF	CTATACTTTAACGTCAAGGAGCCGCGGATGGCAAACCCTTTTTCGAG
Aur1GR	GGGAGACCGGCAGATGCCGCTCTAGACCTAATAAC
ADH1F	CTAGTGGCCTATGCGGCCGCGGATCCTTTTGTTGTTTCCGGG
ADH1R	GGGAGACCGGCAGATCCGCGGCCGGTAGAGGTGTGGTCAATAAG

PKCbetaF	GGATGAATTGTACAAAGGATCCAtgGtgcatgaggtcaagaac
PKCbetaR	ATATGGCGCGCCTCACCTAGGCTAgcgctccgtgtggtccgtgc
APEX2-Clhn-F	GGGTTAATTAACATCGCTAGCGGAAAGTCTTACCCAACTGTG
Clhn-APEX2-R	GTGGCGCGCCTCACCTAGGCTAAGATCTGCACTGAGCGTC
